# Supplementary material for: Coordination of Pickpocket ion channel delivery and dendrite growth in Drosophila sensory neurons
Source: PLoS Genet. 2023 Nov 9;19(11):e1011025. doi: 10.1371/journal.pgen.1011025 (PMC10662761; doi:10.1371/journal.pgen.1011025)
Supplement: S1 Fig — Representative images and quantification of membrane-expressed Ppk1, recognized by anti Ppk1 antibodies (top) and sfGFP-tagged Ppk1 (bottom) in control neurons (w1118;12 larvae, 36 neurons) and neurons heterozygous for Ppk1 tagged at the N- or C-terminus (sfGFP::Ppk1 and Ppk1:: sfGFP, respectively) (sfGFP::ppk1; 11 larvae, 33 neurons and ppk1:: sfGFP; 11 larvae, 33 neurons). Quantification, Ppk1 membrane levels (top graph): One-way ANOVA with post-hoc Tukey: control v. sfGFP::ppk1 (p = 0.2570), control v. ppk1::sfGFP (p = 0.9097), ppk1::sfGFP v. sfGFP::ppk1 (p = 0.4802). Quantification, sfGFP-tagged Ppk1 (bottom graph): Student’s unpaired t-test (p = 0.1707). In the graphs, each data point represents the average signal intensity per larva (2–3 neurons per larva). Data are plotted as mean± SEM. n.s. = not significant (p>0.05). AU: arbitrary units. Scale bar, 50 μm. (PDF) [file pgen.1011025.s001.pdf]

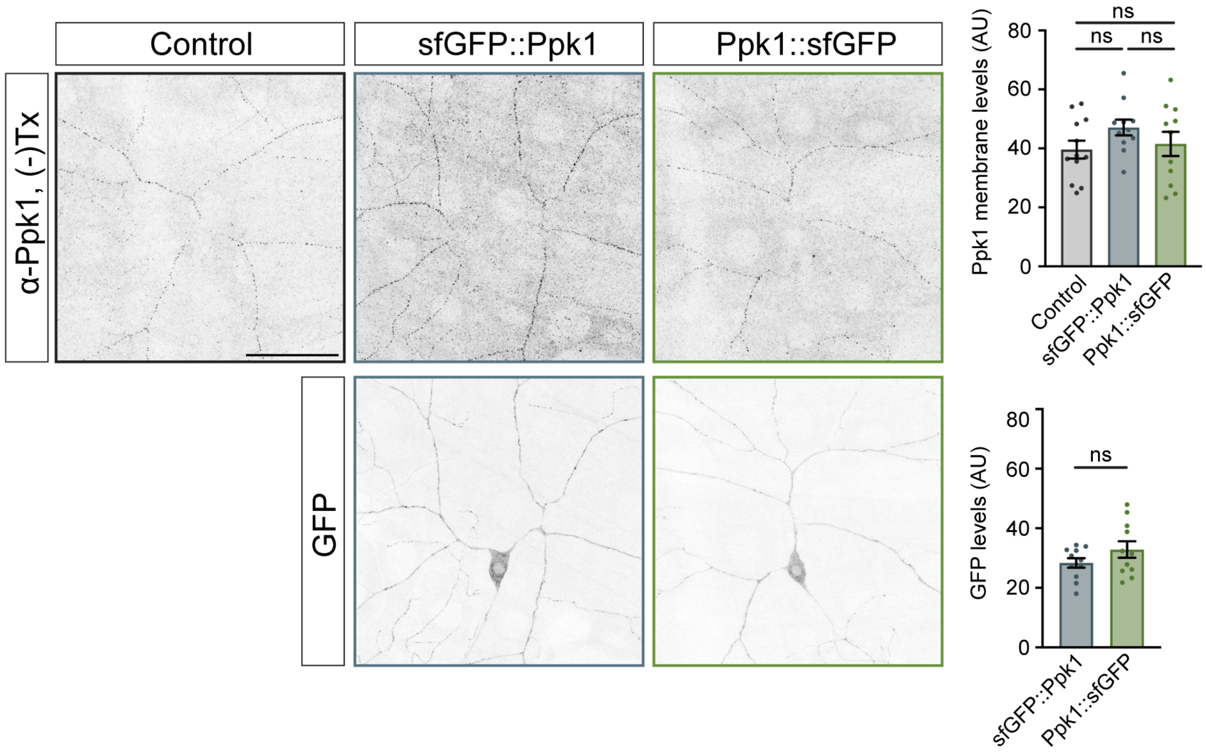

S1 Fig. Effects of tagging endogenous Ppk1 on Ppk1 levels.
Representative images and quantification of membrane-expressed Ppk1, recognized by anti-Ppk1 antibodies (top) and sfGFP-tagged Ppk1 (bottom) in control neurons (*w^{1118}*;12 larvae, 36 neurons) and neurons heterozygous for Ppk1 tagged at the N- or C-terminus (sfGFP::Ppk1 and Ppk1::sfGFP, respectively) (*sfGFP::ppk1*; 11 larvae, 33 neurons and *ppk1::sfGFP*; 11 larvae, 33 neurons). Quantification, Ppk1 membrane levels (top graph): One-way ANOVA with post-hoc Tukey: control v. *sfGFP::ppk1* (p=0.2570), control v. *ppk1::sfGFP* (p=0.9097), *ppk1::sfGFP* v. *sfGFP::ppk1* (p=0.4802). Quantification, sfGFP-tagged Ppk1 (bottom graph): Student's unpaired t-test (p=0.1707). In the graphs, each data point represents the average signal intensity per larva (2-3 neurons per larva). Data are plotted as mean ± SEM. n.s.=not significant (p>0.05). AU: arbitrary units. Scale bar, 50 μm.
