# Supplementary material for: Coordination of Pickpocket ion channel delivery and dendrite growth in Drosophila sensory neurons
Source: PLoS Genet. 2023 Nov 9;19(11):e1011025. doi: 10.1371/journal.pgen.1011025 (PMC10662761; doi:10.1371/journal.pgen.1011025)
Supplement: S2 Fig — Representative images of ddaC neurons in live 3rd instar larvae (A, B, D-E) and axon terminals in fixed ventral nerve cords (VNCs) (C). Dashed-outline boxes: zoomed-in views of dendrite branch and cell bodies. (A) Representative image of Ppk1::mCherry. Dashed-outline boxes: Individual 1-μm thick z-plane zoomed in views of dendrites and axons; a line indicates the position at which an intensity profile plot was generated. Scale bars, 50 μm and 5 μm (dashedoutline boxes). (B) Representative images of the cell body and proximal dendrites of Ppk1::sfGFP, Ppk1::mCherry, and sfGFP::Ppk1::mCherry. Scale bar, 10 μm. (C) Representative images of dual-tagged sfGFP::Ppk1::mCherry in dendrites, cell body, proximal axon, and axon terminals in the VNC. Scale bars, 50 μm (left), 5 μm (middle dashed-outline boxes), 10 μm (right). (D) Representative images of Ppk1::mCherry relative to Rab5::GFP (top) and Rab7::GFP (bottom). Arrowheads: Colocalized signal. Scale bars, 10 μm. (E) Representative images and quantification of Ppk1::mCherry in control (20 neurons, 10 larvae) and Rab5-DN-expressing neurons (20 neurons, 10 larvae). Quantification, Ppk1::mCherry puncta number: Mann-Whitney test (p<0.0001). Ppk1::mCherry puncta were quantified in dendrites within 70 μm of the cell body. Control genotype: w1118; ppk-Gal4. Experimental genotype: w1118; ppk-Gal4 UAS-Rab5-DN::YFP (E). UAS-Rab5::GFP, UAS-Rab7::GFP, and ppk1::mCherry included as indicated (D-E). Scale bar 10 μm. In the graphs, each data point represents a neuron, and data are plotted as mean ±SEM. ****p<0.0001. AU: arbitrary units. (PDF) [file pgen.1011025.s002.pdf]

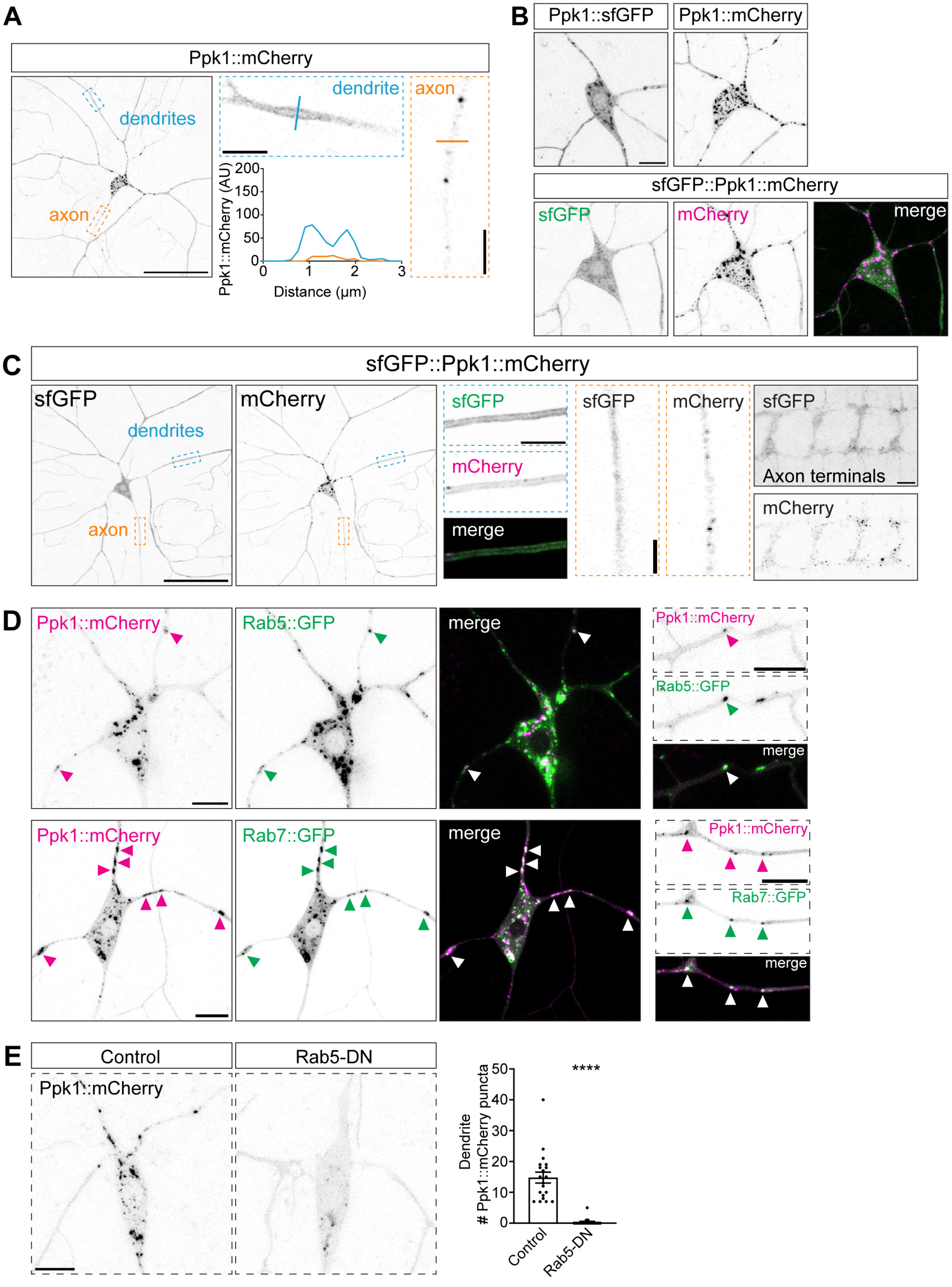

A
Ppk1::mCherry
dendrites
axon
dendrite
axon
Ppk1::mCherry (AU)
200
150
100
50
0
0
1
2
3
Distance (μm)
B
Ppk1::sfGFP
Ppk1::mCherry
sfGFP::Ppk1::mCherry
sfGFP
mCherry
merge
C
sfGFP::Ppk1::mCherry
sfGFP
mCherry
dendrites
axon
sfGFP
mCherry
merge
sfGFP
mCherry
sfGFP
Axon terminals
mCherry
D
Ppk1::mCherry
Rab5::GFP
merge
Ppk1::mCherry
Rab5::GFP
merge
Ppk1::mCherry
Rab7::GFP
merge
Ppk1::mCherry
Rab7::GFP
merge
E
Control
Rab5-DN
Ppk1::mCherry
Dendrite
# Ppk1::mCherry puncta
50
40
30
20
10
0
****
Control
Rab5-DN

**S2 Fig. Localization of Ppk1 tagged with mCherry.**

Representative images of ddaC neurons in live 3rd instar larvae (A, B, D-E) and axon terminals in fixed ventral nerve cords (VNCs) (C). Dashed-outline boxes: zoomed-in views of dendrite branch and cell bodies. (A) Representative image of Ppk1::mCherry. Dashed-outline boxes: Individual 1-μm thick z-plane zoomed in views of dendrites and axons; a line indicates the position at which an intensity profile plot was generated. Scale bars, 50 μm and 5 μm (dashed-outline boxes). (B) Representative images of the cell body and proximal dendrites of Ppk1::sfGFP, Ppk1::mCherry, and sfGFP::Ppk1::mCherry. Scale bar, 10 μm. (C) Representative images of dual-tagged sfGFP::Ppk1::mCherry in dendrites, cell body, proximal axon, and axon terminals in the VNC. Scale bars, 50 μm (left), 5 μm (middle dashed-outline boxes), 10 μm (right). (D) Representative images of Ppk1::mCherry relative to Rab5::GFP (top) and Rab7::GFP (bottom). Arrowheads: Colocalized signal. Scale bars, 10 μm. (E) Representative images and quantification of Ppk1::mCherry in control (20 neurons, 10 larvae) and Rab5-DN-expressing neurons (20 neurons, 10 larvae). Quantification, Ppk1::mCherry puncta number: Mann-Whitney test ($p<0.0001$). Ppk1::mCherry puncta were quantified in dendrites within 70 μm of the cell body. Control genotype: $w^{1118}$; *ppk-Gal4*. Experimental genotype: $w^{1118}$; *ppk-Gal4 UAS-Rab5-DN::YFP* (E). *UAS-Rab5::GFP*, *UAS-Rab7::GFP*, and *ppk1::mCherry* included as indicated (D-E). Scale bar 10 μm. In the graphs, each data point represents a neuron, and data are plotted as mean ±SEM. ****$p<0.0001$. AU: arbitrary units.
