# Supplementary material for: Coordination of Pickpocket ion channel delivery and dendrite growth in Drosophila sensory neurons
Source: PLoS Genet. 2023 Nov 9;19(11):e1011025. doi: 10.1371/journal.pgen.1011025 (PMC10662761; doi:10.1371/journal.pgen.1011025)
Supplement: S3 Fig — (A) Representative images of ddaC neurons expressing Ppk1 tagged with one copy of sfGFP at two different extracellular (EC) sites, Site 1 (between Asn171 and lle172) and Site 2 (between Gln204 and Leu205). Cartoon (left) shows the crystal structure of an individual cASIC1 subunit (PDB: 2QTS)[18]. Ppk1 is predicted to have a similar structure); three subunits compose a channel. The amino acid sequence for D. melanogaster is superimposed on the cASIC cartoon structure, and the sites that correspond to where sfGFP was inserted into Ppk1 are indicated. The locations of Site 1 and Site 2 were predicted by aligning the amino acid sequences of Ppk1 and cASIC1. Ppk1 tagged with sfGFP at Site 1 showed similar fluorescent signal as Ppk1 tagged with sfGFP at the N- or Cterminus; thus, Site 1 was used for the insertion of additional tags (e.g., superecliptic pHluorin and GFP(11)). Site 2 is located near the position at which a haemagglutinin (HA) tag was inserted in rASIC1a [32]. (B) Representative image of a ddaC neuron expressing Ppk1 tagged with one copy of pHluorin at Site 1 and mCherry at the C-terminus (Ppk1:: pHluorinEC::mCherryC-term). The neuron is heterozygous for ppk1:: pHluorinEC::mCherryC-term. Scale bar, 50 μm. (C) Representative images of ddaC neurons expressing Ppk1 tagged with one copy of pHluorin at Site 1 and mScarlet at the C-terminus (Ppk1::pHluorinEC::mScarletC-term) in control neurons (w1118) and neurons lacking ppk26 (11ppk26: ppk26/J111M1). The neurons are homozygous for ppk1:: pHluorinEC::mScarletC-term. Scale bar, 50 μm. (PDF) [file pgen.1011025.s003.pdf]

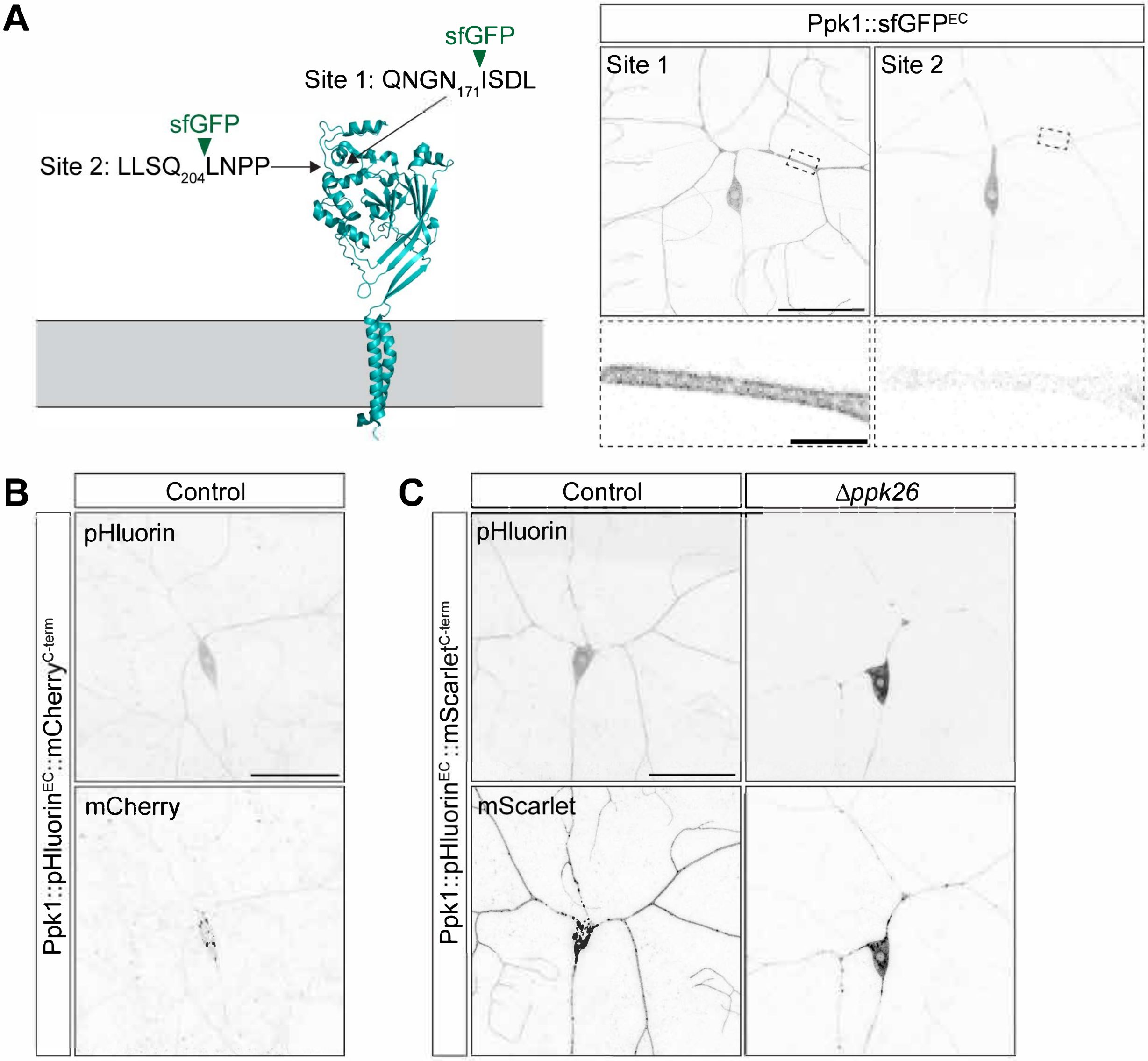

S3 Fig. Endogenous Ppk1 tagged with sfGFP and pHluorin at extracellular positions.
(A) Representative images of ddaC neurons expressing Ppk1 tagged with one copy of sfGFP at two different extracellular (EC) sites, Site 1 (between Asn171 and Ile172) and Site 2 (between Gln204 and Leu205). Cartoon (left) shows the crystal structure of an individual cASIC1 subunit (PDB: 2QTS)[18]. Ppk1 is predicted to have a similar structure); three subunits compose a channel. The amino acid sequence for *D. melanogaster* is superimposed on the cASIC cartoon structure, and the sites that correspond to where sfGFP was inserted into Ppk1 are indicated. The locations of Site 1 and Site 2 were predicted by aligning the amino acid sequences of Ppk1 and cASIC1. Ppk1 tagged with sfGFP at Site 1 showed similar fluorescent signal as Ppk1 tagged with sfGFP at the N- or C-terminus; thus, Site 1 was used for the insertion of additional tags (e.g., superecliptic pHluorin and GFP(11)). Site 2 is located near the position at which a haemagglutinin (HA) tag was inserted in rASIC1a [32]. (B) Representative image of a ddaC neuron expressing Ppk1 tagged with one copy of pHluorin at Site 1 and mCherry at the C-terminus (Ppk1::pHluorinEC::mCherryC-term). The neuron is heterozygous for ppk1::pHluorinEC::mCherryC-term. Scale bar, 50 μm. (C) Representative images of ddaC neurons expressing Ppk1 tagged with one copy of pHluorin at Site 1 and mScarlet at the C-terminus (Ppk1::pHluorinEC::mScarletC-term) in control neurons (w^{1118}) and neurons lacking *ppk26* (*Δppk26*: $ppk26^{Δ11/Δ11}$). The neurons are homozygous for ppk1::pHluorinEC::mScarletC-term. Scale bar, 50 μm.
