# Supplementary material for: Coordination of Pickpocket ion channel delivery and dendrite growth in Drosophila sensory neurons
Source: PLoS Genet. 2023 Nov 9;19(11):e1011025. doi: 10.1371/journal.pgen.1011025 (PMC10662761; doi:10.1371/journal.pgen.1011025)
Supplement: S4 Fig — Representative images of ddaC neurons in live 3rd instar larvae. (A) By itself, secGFP(1–10) is not fluorescent (DcG-Ga/4 UAS-secGFP(1–10)) (top row). GFP(11) is not fluorescent in the absence of GFP(1–10) (ppk1:: GFP(11x3)Ec::mCher,yG-1erm)(middle row). The expression of GFP(1–10) in neurons does not result in fluorescence, consistent with the model that GFP(11), located on an extracellular loop of Ppk1, is not exposed to the cytoplasm where it might encounter GFP(1–10) (ppk Ga/4 UAS-GFP(1–10) in combination with ppk1::GFP(11x3)Ec::mCher,yc-1erm; the ddaC neurons also express CD4::tdTomato under the control of a ppk enhancer, ppk-CD4::tdTomato)(bottom row). (B) By itself, GFP(11) Ec:: CD4::tdTomato does not produce any GFP fluorescence (left). In the presence of secGFP(1–10), GFP(11)Ec::CD4::tdTomato, fluorescent GFP is visible throughout the ddaC neuron (right). GFP(11) Ec:: CD4::tdTomato is expressed in class IV da neurons under the control of a ppk enhancer, ppk-GFP(11)Ec::CD4::tdTomato. Black arrowheads point to the cell body. Scale bar, 50 μm. (PDF) [file pgen.1011025.s004.pdf]

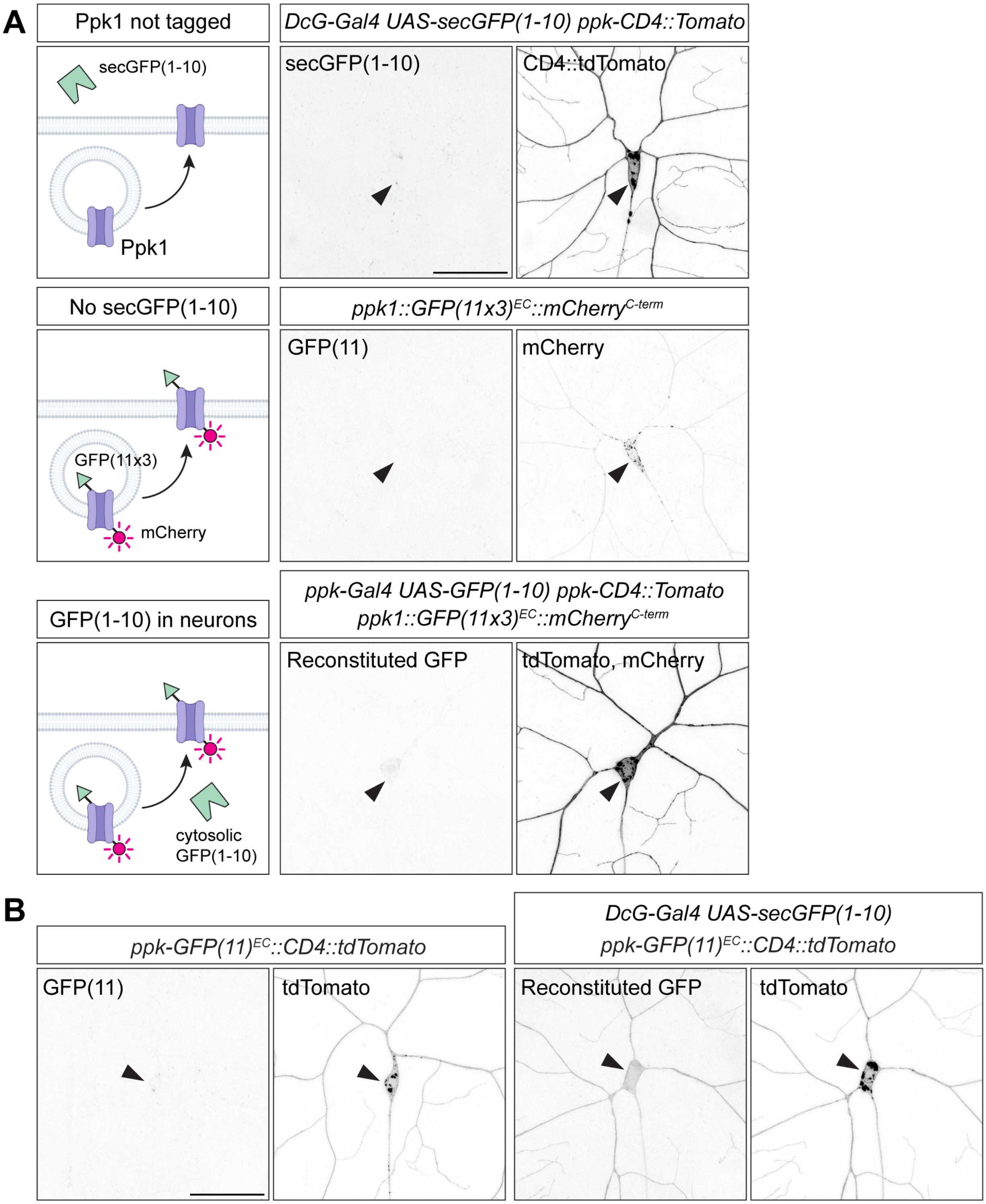

S4 Fig. Characterization of a split-GFP-based approach to label membrane-expressed Ppk1. Representative images of ddaC neurons in live 3rd instar larvae. (A) By itself, secGFP(1-10) is not fluorescent (*DcG-Gal4 UAS-secGFP(1-10)*) (top row). GFP(11) is not fluorescent in the absence of GFP(1-10) (*ppk1::GFP(11x3)^{EC}::mCherry$^{C\text{-}term}$*)(middle row). The expression of GFP(1-10) in neurons does not result in fluorescence, consistent with the model that GFP(11), located on an extracellular loop of Ppk1, is not exposed to the cytoplasm where it might encounter GFP(1-10) (*ppk-Gal4 UAS-GFP(1-10)* in combination with *ppk1::GFP(11x3)^{EC}::mCherry$^{C\text{-}term}$*; the ddaC neurons also express CD4::tdTomato under the control of a *ppk* enhancer, *ppk-CD4::tdTomato*)(bottom row). (B) By itself, GFP(11)^{EC}::CD4::tdTomato does not produce any GFP fluorescence (left). In the presence of secGFP(1-10), GFP(11)^{EC}::CD4::tdTomato, fluorescent GFP is visible throughout the ddaC neuron (right). GFP(11)^{EC}::CD4::tdTomato is expressed in class IV da neurons under the control of a *ppk* enhancer, *ppk-GFP(11)^{EC}::CD4::tdTomato*. Black arrowheads point to the cell body. Scale bar, 50 μm.
