# Supplementary material for: Coordination of Pickpocket ion channel delivery and dendrite growth in Drosophila sensory neurons
Source: PLoS Genet. 2023 Nov 9;19(11):e1011025. doi: 10.1371/journal.pgen.1011025 (PMC10662761; doi:10.1371/journal.pgen.1011025)
Supplement: S5 Fig — For each dendrite growth mutant, the data are plotted as mean± SEM; n represents number of neurons. w1118 is the control genotype. Quantification, Sholl analysis (mean± SEM): Critical radius (μm), control = 127 ± 3, n = 12, and Rp/22-RNAi = 124 ± 6, n = 13, Student’s unpaired t-test (p = 0.9467); control = 131 ± 4, n = 15, and EcR-DN = 138 ± 4, n = 15, Student’s unpaired t-test (p = 0.2475); control = 146 ± 5, n = 15, and Rac1 O/E = 82 ± 4, n = 14, Student’s unpaired t-test (p<0.0001); control = 132 ± 5, n = 12, and P/3K O/E = 130 ± 4, n = 12, Student’s unpaired t-test (p = 0.7345). Maximum number of intersections, control = 38 ± 2, n = 12, and Rp/22-RNAi = 20 ± 1, n = 13, Student’s unpaired t-test (p<0.0001); control = 41 ± 1, n = 15, and EcR-DN = 29 ± 1, n = 15, Student’s unpaired t-test (p<0.0001); control = 42 ± 1, n = 15, and Rac1 O/E = 52 ± 2, n = 14, Student’s unpaired t-test (p<0.0001); control = 47 ± 2, n = 12, and P/3K O/E = 58 ± 2, n = 12, Student’s unpaired t-test (p = 0.0002). (PDF) [file pgen.1011025.s005.pdf]

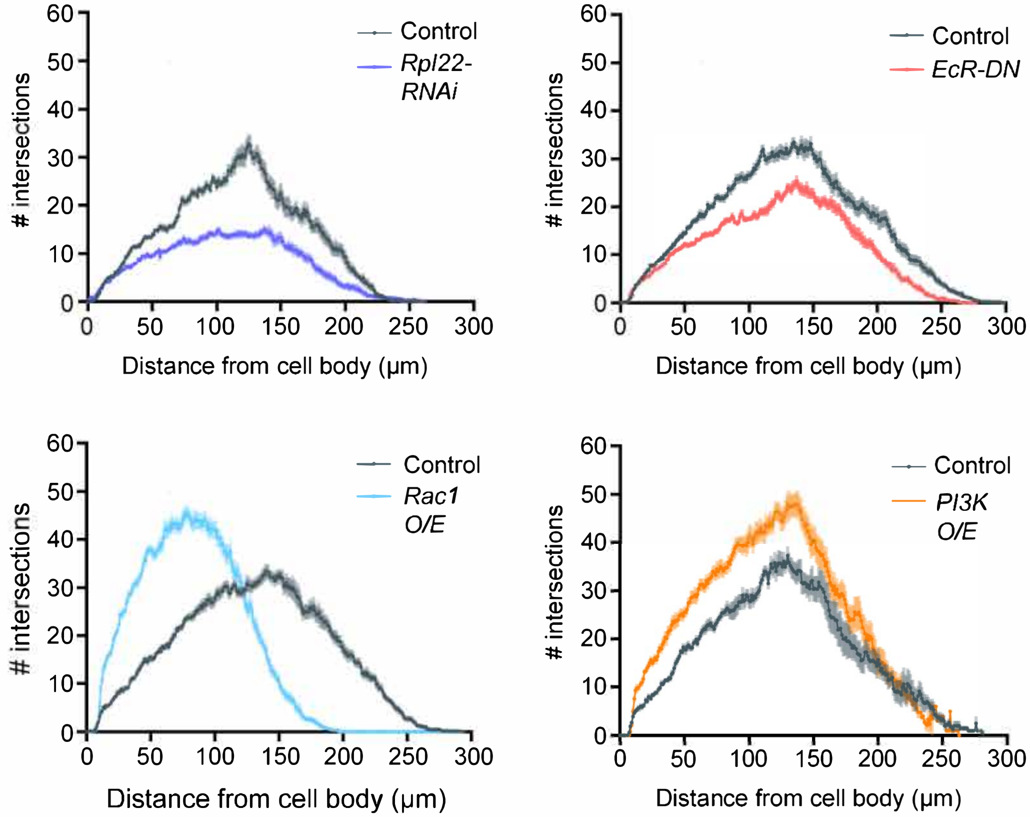

S5 Fig. Sholl analysis of dendritic arbors in dendrite growth mutants.

For each dendrite growth mutant, the data are plotted as mean ± SEM; n represents number of neurons. w^{1118} is the control genotype. Quantification, Sholl analysis (mean ± SEM): Critical radius (μm), control = 127 ± 3, n=12, and *Rpl22-RNAi* = 124 ± 6, n=13, Student's unpaired t-test (p=0.9467); control = 131 ± 4, n=15, and *EcR-DN* = 138 ± 4, n=15, Student's unpaired t-test (p=0.2475); control = 146 ± 5, n=15, and *Rac1 O/E* = 82 ± 4, n=14, Student's unpaired t-test (p<0.0001); control = 132 ± 5, n=12, and *PI3K O/E* = 130 ± 4, n=12, Student's unpaired t-test (p=0.7345). Maximum number of intersections, control = 38 ± 2, n=12, and *Rpl22-RNAi* = 20 ± 1, n=13, Student's unpaired t-test (p<0.0001); control = 41 ± 1, n=15, and *EcR-DN* = 29 ± 1, n=15, Student's unpaired t-test (p<0.0001); control = 42 ± 1, n=15, and *Rac1 O/E* = 52 ± 2, n=14, Student's unpaired t-test (p<0.0001); control = 47 ± 2, n=12, and *PI3K O/E* = 58 ± 2, n=12, Student's unpaired t-test (p=0.0002).
