# Supplementary material for: Coordination of Pickpocket ion channel delivery and dendrite growth in Drosophila sensory neurons
Source: PLoS Genet. 2023 Nov 9;19(11):e1011025. doi: 10.1371/journal.pgen.1011025 (PMC10662761; doi:10.1371/journal.pgen.1011025)
Supplement: S6 Fig — Representative images of ddaC neurons in live 3rd instar larvae. The dendritic membrane is marked by CD4::tdTomato. Dashed-outline boxes: Zoomed-in views of dendrite branches. Representative images of control larvae expressing endogenous TrpA1 tagged at the N-terminus with GFP (GFP::TrpA1) (top left). Representative image and quantification of EcR-DN-expressing neurons (top right). Quantification, GFP::TrpA1, dendrites: Student’s unpaired t-test (p<0.0001); control (26 neurons, 11 larvae) v. EcR-DN (24 neurons, 10 larvae). Representative image and quantification of neurons over expressing Rac1 (bottom left). Quantification, GFP::TrpA1, dendrites: Student’s unpaired t-test (p<0.0001); control (26 neurons, 11 larvae) v. Rac1 O/E (22 neurons, 11 larvae). The same control was used for both EcR-DN and Rac1 O/E (experiments were carried out in parallel). Control genotype: ppk-Gal4 ppk CD4::tdTomato; GFP::TrpA1. Experimental genotypes: ppk-Gal4 ppk-CD4::tdTomato/UAS-EcR-DN; GFP::TrpA1 and ppk-Gal4 ppk-CD4::tdTomato/UAS-Rac1; GFP::TrpA1. Scale bars, 50 μm and 10 μm (dashed outline boxes). (PDF) [file pgen.1011025.s006.pdf]

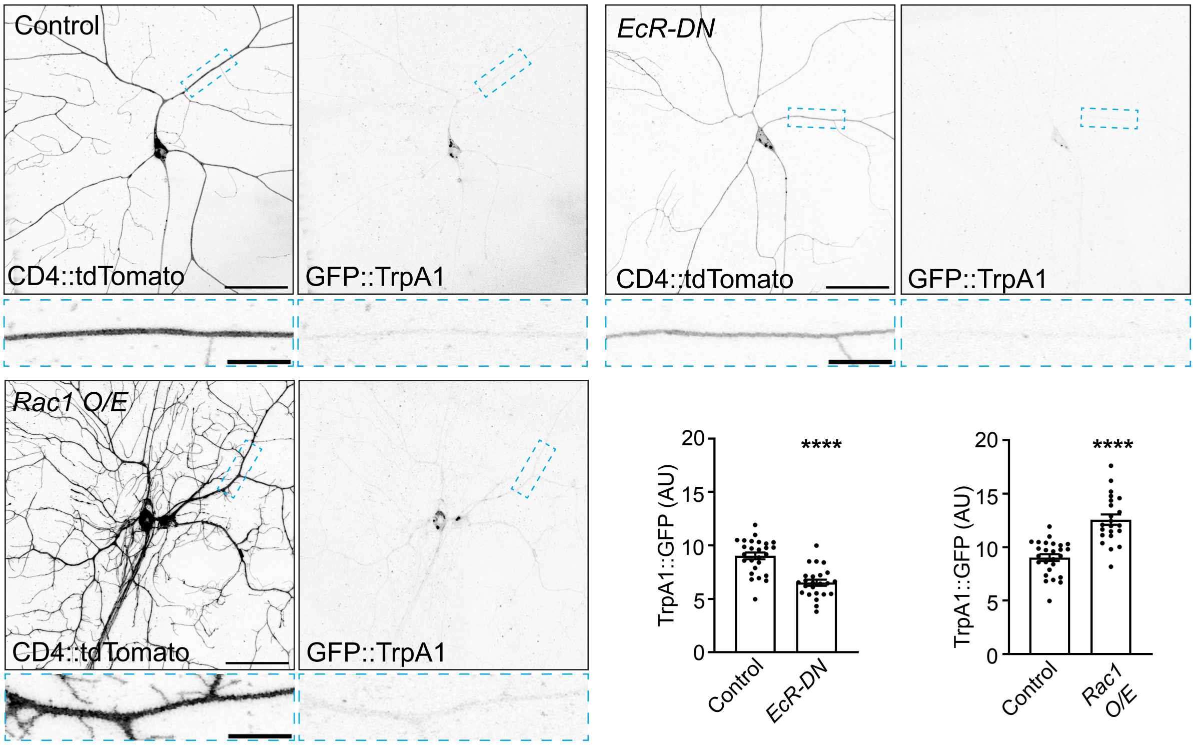

S6 Fig. Effects of altering dendritic growth on GFP::TrpA1.
Representative images of ddaC neurons in live 3rd instar larvae. The dendritic membrane is marked by CD4::tdTomato. Dashed-outline boxes: Zoomed-in views of dendrite branches. Representative images of control larvae expressing endogenous TrpA1 tagged at the N-terminus with GFP (*GFP::TrpA1*) (top left). Representative image and quantification of *EcR-DN*-expressing neurons (top right). Quantification, GFP::TrpA1, dendrites: Student's unpaired t-test ($p<0.0001$); control (26 neurons, 11 larvae) v. *EcR-DN* (24 neurons, 10 larvae). Representative image and quantification of neurons over-expressing *Rac1* (bottom left). Quantification, GFP::TrpA1, dendrites: Student's unpaired t-test ($p<0.0001$); control (26 neurons, 11 larvae) v. *Rac1 O/E* (22 neurons, 11 larvae). The same control was used for both *EcR-DN* and *Rac1 O/E* (experiments were carried out in parallel). Control genotype: *ppk-Gal4 ppk-CD4::tdTomato; GFP::TrpA1*. Experimental genotypes: *ppk-Gal4 ppk-CD4::tdTomato/UAS-EcR-DN*; *GFP::TrpA1* and *ppk-Gal4 ppk-CD4::tdTomato/UAS-Rac1; GFP::TrpA1*. Scale bars, 50 μm and 10 μm (dashed-outline boxes).
