# Supplementary material for: Coordination of Pickpocket ion channel delivery and dendrite growth in Drosophila sensory neurons
Source: PLoS Genet. 2023 Nov 9;19(11):e1011025. doi: 10.1371/journal.pgen.1011025 (PMC10662761; doi:10.1371/journal.pgen.1011025)
Supplement: S7 Fig — Representative Images of ddaC neurons in live 3rd instar larvae. Dashed-outline boxes: Zoomed-in views of axons. (A) Representative images and quantification of Ppk1::sfGFP in control (21 neurons, 11 larvae) and Dlic-RNAi-expressing neurons (21 neurons, 11 larvae). Quantification, axons: Mann-Whitney test (p<0.0001). Scale bars, 50 μm and 5 μm (dashed-outline boxes). Representative images and quantification of Ppk1::sfGFP in control neurons and neurons over-expressing dmn (O/E dmn). Quantification, axons: Mann-Whitney test (p<0.0001); control (20 neurons, 12 larvae) v. O/E dmn (23 neurons, 14 larvae). Scale bars, 50 μm and 5 μm (dashed-outline boxes). (B) Representative images of Ppk1::sfGFP and GALNT2::TagRFP in the axons of control and Dlic-RNAi-expressing neurons. Zoomed in images of an axon of a Dlic-RNAi-expressing neuron shows that the Ppk1::sfGFP puncta (green arrowheads) do not colocalize with ectopic Golgi marked by GALNT2::TagRFP (magenta arrowheads). Scale bars, 10 μm and 5 μm (dashed-outline boxes). (C) Representative images of GALNT2::TagRFP and Ppk1::sfGFP in the axons of control neurons and neurons expressing a dominant-negative form of the Golgin Lava lamp (Lva) (Lva-DN); Lva-DN disrupts the interaction between Golgi and dynein but does not affect dynein activity. In neurons expressing lva-DN, GALNT2::TagRFP mislocalizes to axons (black arrowheads) and axonal Ppk1::sfGFP levels increase. Quantification, Ppk1::sfGFP, axons: Student’s unpaired t-test (p<0.0001); control (17 neurons, 6 larvae) v. lva-DN (16 neurons, 7 larvae). Scale bar, 10 μm. Control genotypes: w1118; ppk-Gal4. Experimental genotypes: w1118; ppk-Gal4 UAS-Dlic-RNAi UAS-Dicer (A and B), w1118; ppk-Gal4 UAS-dmn (A), w1118; ppk-Gal4 UAS-lva-DN (C). UAS-GALNT2::TagRFP and ppk1::sfGFP included as indicated (A-C). In the graphs, each data point represents a neuron and data are plotted as mean ± SEM. ****p<0.0001. AU: arbitrary units. (PDF) [file pgen.1011025.s007.pdf]

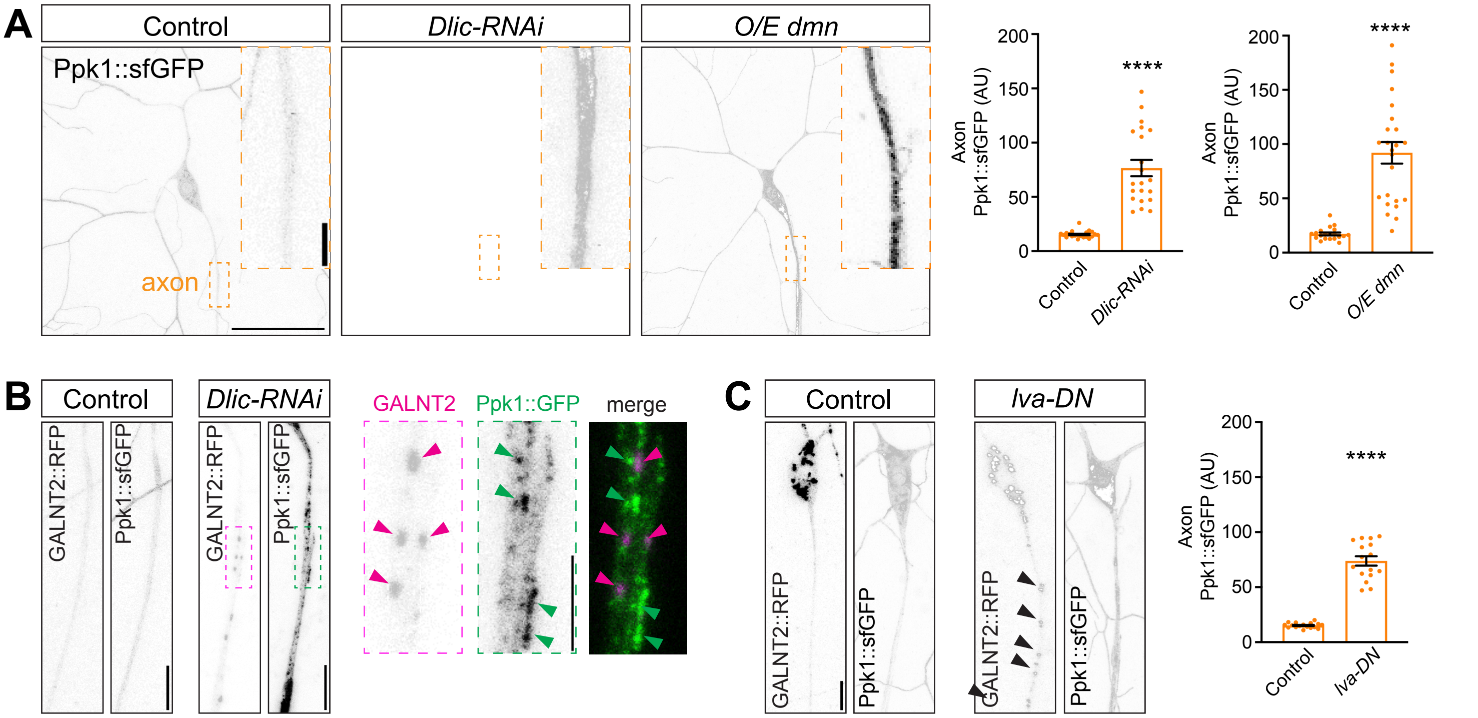

S7 Fig. Ppk1 localizes to axons when dynein-mediated transport is disrupted.
Representative Images of ddaC neurons in live 3rd instar larvae. Dashed-outline boxes: Zoomed-in views of axons. (A) Representative images and quantification of Ppk1::sfGFP in control (21 neurons, 11 larvae) and *Dlic-RNAi*-expressing neurons (21 neurons, 11 larvae). Quantification, axons: Mann-Whitney test ($p<0.0001$). Scale bars, 50 µm and 5 µm (dashed-outline boxes). Representative images and quantification of Ppk1::sfGFP in control neurons and neurons over-expressing *dmn* (*O/E dmn*). Quantification, axons: Mann-Whitney test ($p<0.0001$); control (20 neurons, 12 larvae) v. *O/E dmn* (23 neurons, 14 larvae). Scale bars, 50 µm and 5 µm (dashed-outline boxes). (B) Representative images of Ppk1::sfGFP and GALNT2::TagRFP in the axons of control and *Dlic-RNAi*-expressing neurons. Zoomed-in images of an axon of a *Dlic-RNAi*-expressing neuron shows that the Ppk1::sfGFP puncta (green arrowheads) do not colocalize with ectopic Golgi marked by GALNT2::TagRFP (magenta arrowheads). Scale bars, 10 µm and 5 µm (dashed-outline boxes). (C) Representative images of GALNT2::TagRFP and Ppk1::sfGFP in the axons of control neurons and neurons expressing a dominant-negative form of the Golgin Lava lamp (Lva) (Lva-DN); Lva-DN disrupts the interaction between Golgi and dynein but does not affect dynein activity. In neurons expressing *lva-DN*, GALNT2::TagRFP mislocalizes to axons (black arrowheads) and axonal Ppk1::sfGFP levels increase. Quantification, Ppk1::sfGFP, axons: Student's unpaired t-test ($p<0.0001$); control (17 neurons, 6 larvae) v. *lva-DN* (16 neurons, 7 larvae). Scale bar, 10 µm. Control genotypes: *w^{1118}; ppk-Gal4*. Experimental genotypes: *w^{1118}; ppk-Gal4 UAS-Dlic-RNAi UAS-Dicer* (A and B), *w^{1118}; ppk-Gal4 UAS-dmn* (A), *w^{1118}; ppk-Gal4 UAS-lva-DN* (C). *UAS-GALNT2::TagRFP* and *ppk1::sfGFP* included as indicated (A-C). In the graphs, each data point represents a neuron and data are plotted as mean ± SEM. ****$p<0.0001$. AU: arbitrary units.
