# Supplementary material for: Coordination of Pickpocket ion channel delivery and dendrite growth in Drosophila sensory neurons
Source: PLoS Genet. 2023 Nov 9;19(11):e1011025. doi: 10.1371/journal.pgen.1011025 (PMC10662761; doi:10.1371/journal.pgen.1011025)
Supplement: S8 Fig — Model illustrating the transport of Ppk channels from the Golgi to dendrites by dynein (left). Our data suggest that this post-Golgi transport involves Rab11, either directly or indirectly (e.g., Ppk channels may be transported to dendrites via Rab11(+) endosomes). When dynein activity is reduced after dendrites have initiated their growth (right), the supply of Ppk channels to dendrites decreases, which culminates in an increase of Ppk channels in the Golgi. Although the localization of Ppk channels to dendrites is reduced when dynein activity decreases, the density of Ppk channels already in the dendritic membrane is not reduced. Created with Biorender.com. (PDF) [file pgen.1011025.s008.pdf]

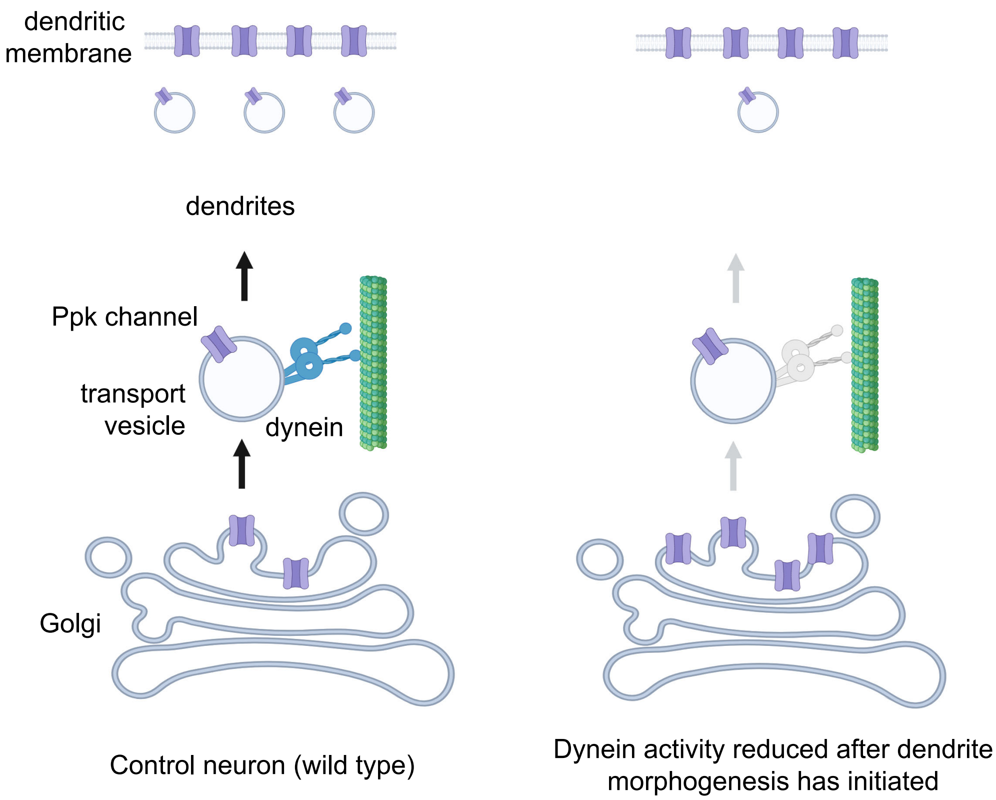

S8 Fig. Dynein implicated in transporting Ppk channels to dendrites. Model illustrating the transport of Ppk channels from the Golgi to dendrites by dynein (left). Our data suggest that this post-Golgi transport involves Rab11, either directly or indirectly (e.g., Ppk channels may be transported to dendrites via Rab11(+) endosomes). When dynein activity is reduced after dendrites have initiated their growth (right), the supply of Ppk channels to dendrites decreases, which culminates in an increase of Ppk channels in the Golgi. Although the localization of Ppk channels to dendrites is reduced when dynein activity decreases, the density of Ppk channels already in the dendritic membrane is not reduced. Created with Biorender.com.
