# Supplementary material for: Coordination of Pickpocket ion channel delivery and dendrite growth in Drosophila sensory neurons
Source: PLoS Genet. 2023 Nov 9;19(11):e1011025. doi: 10.1371/journal.pgen.1011025 (PMC10662761; doi:10.1371/journal.pgen.1011025)
Supplement: Attachment — Submitted filename: Mitchell et al Response to reviewers.pdf [file pgen.1011025.s010.pdf]

Response to Reviewers' Comments and Questions

We thank the reviewers for their thoughtful review of our work. In response to the reviewers' constructive and helpful comments, we have undertaken a major revision of the manuscript. Detailed responses to the reviewers' questions are below. A summary of the major changes to the figures that occurred during the revision can be found at the end of this document.

Reviewer #1: The manuscript by Mitchell et al performed live imaging of a sensory ion channel subunit, Ppk1, in Drosophila peripheral sensory neurons. They showed that Ppk1 was uniformly distributed throughout the dendritic, but not axonal, membrane. Ppk1 levels in the dendrites were not affected by suppression of Rpl22 or EcR, which reduces the dendrite length, suggesting that Ppk1 levels scale in proportion to dendrite length. Their observations also suggest that Rab11 is involved in the trafficking of Ppk1 to the dendrite membrane.
The scaling of sensory ion channels may be important, but the authors analyzed the behavior of one channel protein only and did not provide the molecular mechanisms. Thus, this reviewer felt that this manuscript is too preliminary to publish in PLoS Genet. Specific comments are shown below.

- The authors only analyzed Ppk1, but at least one or two additional sensory ion channels, such as TRP family and Piezo, should be analyzed. Instead of the genome engineering, immunostaining and quantification of TRP and Piezo in the tissue sections used in Fig. 4 are also acceptable.

> This is a great suggestion; however, the reagents to perform these experiments are very limited. Indeed, we feel that the current lack of reagents to monitor endogenous ion channel localization highlights the strength of our new tools to visualize endogenous Ppk1 and Ppk channels.
>
> For Drosophila Piezo, no antibodies are available and the broad of expression Piezo makes it difficult to visualize GFP-tagged endogenous Piezo specifically in the class IV da neurons. The TRP family members expressed in the class IV da neurons include Painless and TrpA1. Painless is expressed at very low levels in da neurons [1], and the anti-Painless antibody very weakly recognizes the channel in dendrites [2]. Endogenous Painless has not be tagged. The anti-TrpA1 antibody does not detect TrpA1 in da neurons, likely because the channel is also expressed at low levels [3]. However, there is a protein-trap strain in which GFP is predicted to be fused at the N-terminus of endogenous TrpA1 (GFP::TrpA1) [4]. Although GFP::TrpA1 is only weakly visible in class IV neurons, the signal is strong enough for quantitative analysis.
>
> We analyzed the density of GFP::TrpA1 in dendrites under two different conditions that disrupt dendrite arborization: the over-expression of Rac1 and expression of a dominant-negative form of the Ecdysone receptor (EcR-DN) (for technical reasons, we were not able to analyze GFP::TrpA1 in the other dendrite growth mutants). Unlike Ppk1::sfGFP, whose density remained constant despite changes in dendrite length and branching, we found that GFP::TrpA1 density changed when dendrite growth was perturbed (new S5 Fig). Overall, these results indicate that Ppk1::GFP density, but not GFP::TrpA1 density, scales with dendrite growth. Thus, the densities of different ion channels in sensory neuron dendrites are likely independently regulated. These new data are included in the revised manuscript (new S5 Fig, see below).

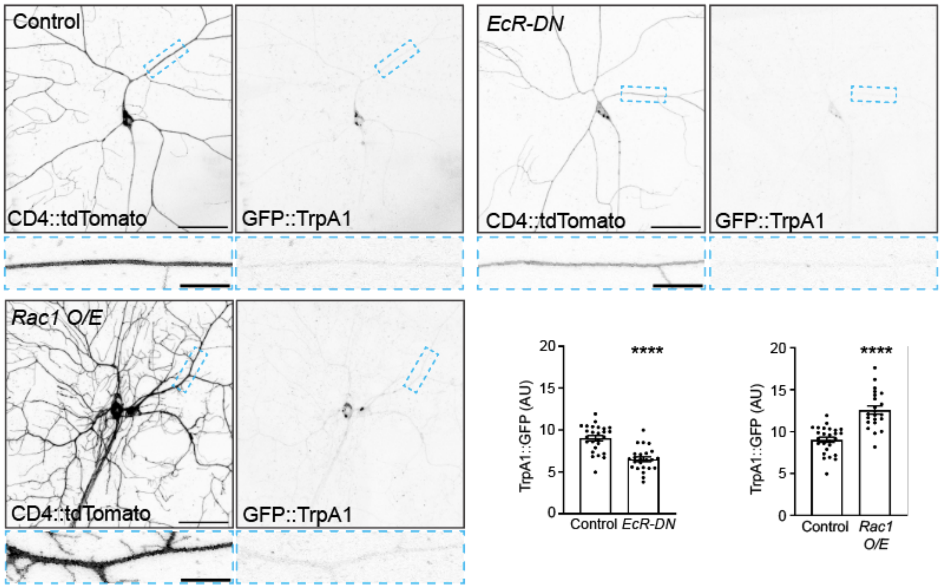

S5 Fig. Effects of EcR-DN and elevated Rac1 on GFP::TrpA1 in dendrites.

New text: "Our finding that Ppk1 density remains constant despite changes in dendrite growth raises the question of whether other ion channels in sensory neuron dendrites might similarly scale in proportion to arbor size. The class IV da neurons also express the TRP channel TrpA1 [17]. To monitor TrpA1 localization and density, we used a protein-trap strain in which GFP is inserted into endogenous TrpA1 near the N-terminus (GFP::TrpA1). Unlike Ppk1::sfGFP, we found that GFP::TrpA1 density fluctuated when dendrite growth was perturbed by the expression of either EcR-DN or elevated Rac1 (S5 Fig). Thus, GFP::TrpA1 does not scale proportionally with changes in dendrite growth, which suggests that ion channels in dendrites are likely differentially regulated."

- A negative control, such as transferrin receptor and N-cadherin, both of which are transported via Rab11 in mammalian neurons, should also be required. Does the suppression of Rpl22 or EcR increase the dendritic density of transferrin receptor and N-cadherin?

We thank the reviewer for this suggestion. While we do not have the tools to visualize endogenous transferrin receptor in Drosophila, and N-cadherin does not appear to be expressed in our model neurons (data not shown), we used antibodies against the fly Neuroglian (Nrg), which others have shown is regulated by Rab11 [5].

The reviewer asks: "Does the suppression of Rpl22 or EcR increase the dendritic density of transferrin receptor and N-cadherin;" however, we believe that the reviewer may have meant "decrease the dendritic density." We believe that the reviewer may be asking whether a Rab11 cargo would be impacted by affecting dendrite growth (we apologize, however, if we are misinterpreting the reviewer's intent). We found that affecting dendrite growth via the expression of either *Rpl22-RNAi* or *EcR-DN* caused a decrease in Nrg in dendrites, consistent with the idea that the growth mutants are affecting Rab11-mediated trafficking. These new data are included below for the reviewer (we have not added these data to the manuscript but will be glad to do so if recommended by the reviewer).

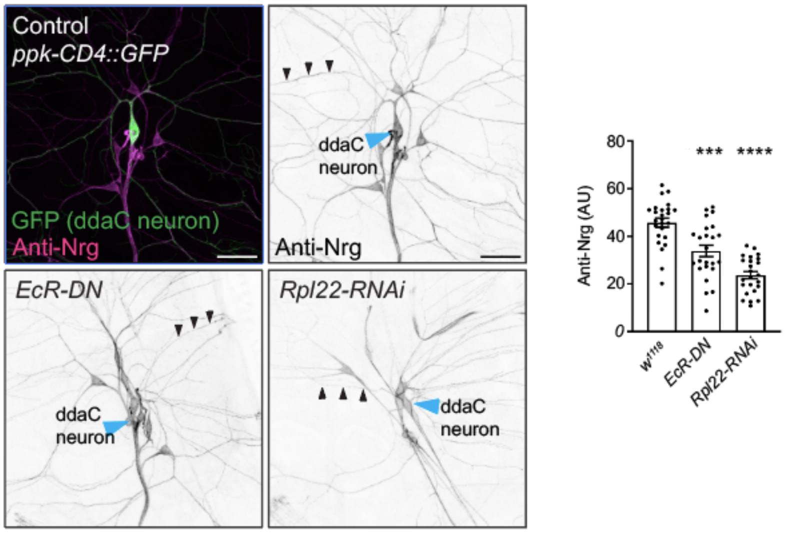

R1 Fig. Disrupting dendrite growth reduces the dendritic density of Nrg.

- Small vesicles may be transported to the growing dendritic membrane to supply membrane lipids. These vesicles (it should be Rab11 positive) would be co-transported with Ppk1 to adjust the same/similar levels of Ppk1 in the dendrite membrane. Stellaris confocal microscopy with HyD is sufficient to measure the ratio of Rab11- and Ppk1-double positive vesicles to Rab11-positive vesicles.

This is an excellent suggestion, and we have attempted these experiments. However, we encountered two difficulties: first, we rarely see Ppk1::sfGFP(+) puncta. It may be that the density of Ppk1::sfGFP in transport vesicles is below our level of detection. Second, both Ppk1 and Rab11 are tagged with GFP, so we are not able to visualize their relative localization. While we have tagged Ppk1 with mCherry, we believe that the majority of the mCherry puncta that we observe reflect Ppk1 in recycling and degradative organelles (new S2 Fig). We have examined the distribution of Ppk1::sfGFP in *Rab11-RNAi*-expressing neurons, and we found that Ppk1::sfGFP accumulates in vesicles/organelles in the cell body when Rab11 is knocked down (new Fig 7D). These new data are included in the manuscript and below.

Although we are not able to image Ppk1 en route to dendrites, we do see Ppk1 in the membrane of actively growing branches, throughout the branch and up to the branch tip, without discernable changes in density (Fig 3B and 3C). This is consistent with the idea that Ppk1 is integral to the membrane that drives arbor expansion.

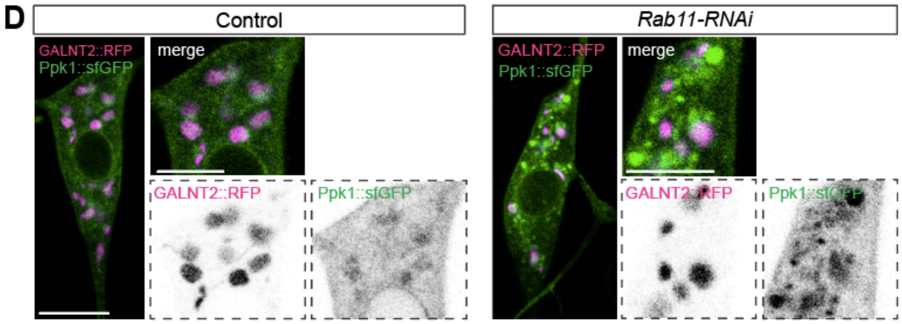

Fig 7D. Disrupting Rab11 alters the distribution of Ppk1::GFP.

- In line with the previous comment, forced ER retention of Ppk1 by addition of KDEL or RXR motif may decrease the supply of vesicle-derived membrane lipids to the dendritic membrane. The authors should examine this.

We thank the reviewer for this suggestion. Our data suggest that the forced ER retention of Ppk1 would not decrease the supply of vesicle-derived membrane lipids to the dendritic membrane. As shown in the original submission, loss of Ppk1 and/or Ppk26 does not affect dendrite growth, which indicates that Ppk channels are not essential for dendrite growth and the delivery of membrane to dendrites (Fig 3E). In the Discussion, we discuss that Ppk channels are not needed for normal dendrite growth.

Reviewer #2: In this manuscript the authors have tried to address the mechanism involved in regulating delivery of ppk1 ion channels in dendritic neurons whether ppk1 trafficking regulates dendrite growth. Using single (sfGFP or mCherry) and double tagged (splitGFP and mCherry) ppk1 coupled to a neat split-GFP technique they show that ppk1 localizes to axonal and dendritic membranes and is also present in organelles. They show that ppk1 is enriched in dendritic membranes compared to axons. Using the split GFP (ppk1 GFP 11) system they demonstrate that a distinct boundary exists between the cell body and the axon which is not observed with CD4 GFP. They find that ppk1 is not essential for growth but channel activity regulates arborization; density of Ppk1 is unaltered despite changes in the length of dendrites; Rab11 and the secretory pathway are involved in membrane localization of Ppk1.

Comments:

1. It is a little confusion at first to understand what the authors mean by 'levels' of Ppk1 in dendrites. One presumes they mean that the density which remains unchanged in response to changes in growth. This should be clarified in the beginning.

> We thank the reviewer for this insightful point. We have revised the text to clarify when we are actually referring to Ppk1 density.

Also, from the different tagged constructs generated, it is evident that the split-GFP method would be the best way to determine membrane localization. However, this system is not used to determine whether there is a change in density of the channel in response of changes in growth or dynein-based transport. Instead, sfGFP tagged Ppk1 is used which would presumable measure cytoplasmic and membrane localized protein.

> We agree with the reviewer that the secreted-split-GFP method we developed is the best way to determine the membrane localization of Ppk1. In the original submission, there was a technical limitation: secGFP(1-10) was expressed in fat bodies via the Gal4-UAS system (*DcG-Gal4 UAS-secGFP(1-10)*). This is an issue because we are also using Gal4-UAS to express constructs to manipulate dendrite growth and dynein-based transport in class IV da neurons; thus, all the UAS constructs (UAS-secGFP(1-10), growth regulators, dynein disrupters) would be expressed in both fat bodies and neurons, creating potential complications in interpreting the results. To circumvent this issue, we created a new construct and fly strains to express secGFP(1-10) in fat bodies under the control of an enhancer active in fat bodies (*R16A03-secGFP(1-10)*). Although three copies of *R16A03-secGFP(1-10)* are needed to obtain sufficient levels of secGFP(1-10) expression for analysis, this new reagent has enabled us to complete some of the suggested experiments.
>
> Using our new *R16A03-secGFP(1-10)* reagent, we analyzed the membrane levels of GFP(11)-tagged Ppk1 when dynein function is disrupted by the expression of *Dlic-RNAi*. Surprisingly, we found a modest but significant increase in the GFP signal intensity of Ppk1::GFP(11x3)^{EC} when dynein function is perturbed. We did not observe any change in Ppk1 membrane levels using an anti-Ppk1 antibody under non-permeabilizing conditions, which suggests that live imaging of Ppk1:GFP(11x3)^{EC} may be a more sensitive readout of Ppk1 membrane localization than the analysis of fixed tissue using anti-Ppk1 (we controlled for the possibility that three copies of GFP(11) may stabilize Ppk1 in the membrane and found no difference in anti-Ppk1 signal between control neurons and neurons expressing Ppk1::GFP(11x3)^{EC}). These new data are included in the revised manuscript (new Fig 6D and 6E) and below.

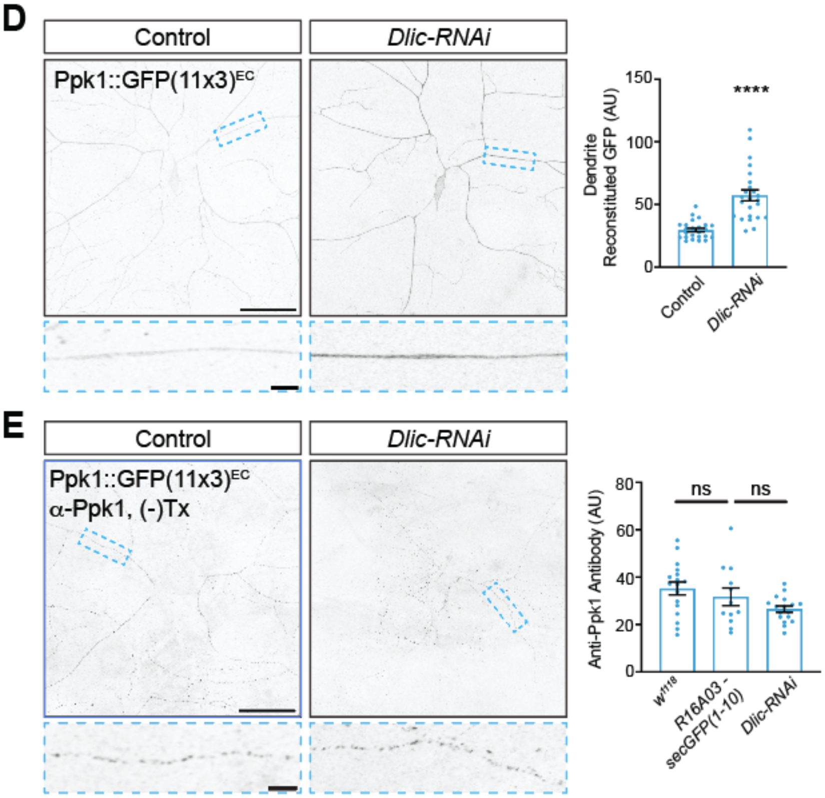

Fig 6. Ppk1 persists in dendrites when dynein-mediated transport is disrupted, and dynein plays a role transporting Ppk1 away from Golgi.

New text: "We also examined the effects of disrupting dynein activity on the dendritic membrane localization of Ppk1 using Ppk1::GFP(11x3)^{EC}. Ppk1::GFP(11x3)^{EC} density increased modestly but significantly in neurons expressing *Dlic-RNAi* (Fig 6D). Given that Ppk1::sfGFP density decreased in *Dlic-RNAi*-expressing neurons, we tested whether the GFP(11x3) tag might affect the stability of Ppk1 in the dendritic membrane. We used an anti-Ppk1 antibody to probe for membrane-localized Ppk1. As reported by anti-Ppk1, the density of membrane-localized Ppk1 was not affected by the GFP(11x3) tag (Fig 6E; the anti-Ppk1 signal is absent in the proximal dendrites, cell body, and axon as these are wrapped by glia, which exclude antibodies under the non-membrane-permeabilizing conditions used to probe for membrane-localized Ppk1 [22]). Also, in neurons expressing *Dlic-RNAi*, the dendritic membrane density of Ppk1 read-out by anti-Ppk1 remained equivalent to control levels (Fig 6E). These results suggest that Ppk1::GFP(11x3)^{EC} may be a more sensitive reporter of Ppk1 membrane localization than the anti-Ppk1 antibody. Our results also suggest that while overall levels of Ppk1 decrease when dynein function is perturbed (e.g., Ppk1::GFP; Fig 6A), Ppk1 that does make it into dendrites and the dendritic membrane is not diminished by reduced dynein activity."

2. Fig. 2E suggests that Δppk1, Δppk26 mutants have increased dendritic branching. What is the effect of ppk1 overexpression on ppk26 degenerin mutant?

First, please note that dendrite branching is normal in *Δppk1 Δppk26* mutants (former Fig 3E, now Fig 5A; in the graph, terminal tip number corresponds to branch number).

Regarding the effect of *ppk1* overexpression on the Ppk26 degenerin-mutant (Ppk26^{DEG}) phenotype: We thank the reviewer for this great suggestion, which has led to the development of an entire new figure (new Fig 5). We overexpressed *ppk1* in neurons expressing Ppk26^{DEG} and found that the elevated levels of Ppk1 partially suppressed the severe degeneration phenotype caused by Ppk26^{DEG} (new Fig 5E). In a control background, approximately half the neurons expressing Ppk26^{DEG} were severely degenerated with dramatically reduced dendritic arbors; indeed, sometimes the neurons were so degenerated that only the cell body and remnants of the dendrites and/or axon remained (Fig 5B). Such severely degenerated neurons were not observed when Ppk1 levels were increased in combination with Ppk26^{DEG} expression (new Fig 5E). These new data are included in the revised manuscript (new Fig 5) and below.

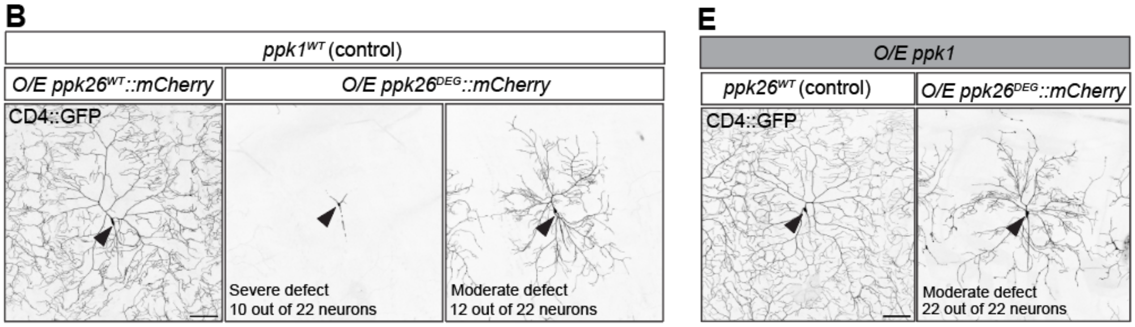

Fig 5. Effects of manipulating Ppk channel expression and activity on dendrite morphogenesis.

3. In their experiment, the authors have examined the role of the secretory pathway in targeting Ppk::GFP to the membrane. Using the double tagged Ppk1::GFP (11x3)Ec::mCherry construct, they show that fluorescence intensity of GFP is significantly reduced whereas levels of mCherry flourescence is only modestly reduced in the membrane. Is it possible that sec23 mutants have less amount of split GFP in the hemolymph? Have the authors looked at Ppk1 levels using immunostaining?

We thank the reviewers for these comments. Please note that *Sec23* was only disrupted in the class IV da neurons and not in the fat body cells that produce secGFP(1-10). *Sec23* was disrupted using a conditional CRISPR-Cas9 approach [6], and Cas9 expression was limited to the class IV da neurons. Therefore, the circulating levels of secGFP(1-10) should not be affected by this approach. We have revised the text to clarify this point.

New text: “We used a transgenic guide RNA (gRNA) that targets the first exon of *Sec23* in combination with a transgene that expresses Cas9 specifically in the class IV da neurons (this limits the disruption of *Sec23* to the class IV da neurons and thus the secretory pathway in other cells is not affected) [28].”

4) Levels of Ppk::sfGFP are unaltered in sec23 mutants compared to controls (Fig. 6). Yet, mcherry fluorescence from Ppk1::GFP (11x3)Ec::mCherry is less in the mutants.
The reason for difference between mCherry tag and sfGFP is not clear. What is the effect of sec23 on localization of Ppk1::mCherry alone?

We thank the reviewer for raising this point. First, in response to this and other reviewer comments on Ppk1::mCherry, we reduced our reliance on mCherry to quantify Ppk1 as the bright fluorescent mCherry puncta make it difficult to accurately quantify the Ppk1::mCherry signal. (Indeed, while revising and reorganizing the manuscript, we removed virtually all quantification of Ppk1 using Ppk1::mCherry.) Second, we repeated the original experiment and, at the same time, as requested by the reviewer, analyzed the effects of disrupting *Sec23* on Ppk1::mCherry alone. When we repeated the experiment, we found no difference between Ppk1::mCherry signal in the control and *Sec23* mutant neurons, regardless of whether the dual- or single-tagged Ppk1 was analyzed (see R2 Fig below). These new data are included below for the reviewer but are not included in the revised manuscript.

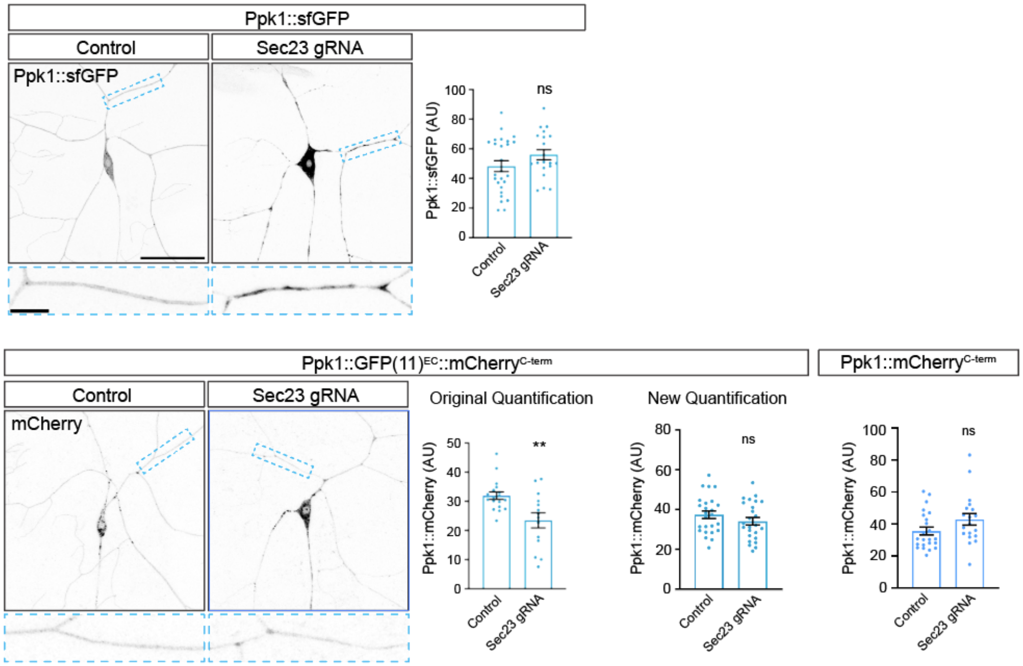

R2 Fig. Disrupting Sec23 does not affect the density of Ppk1 in dendrites as read-out by sfGFP- or mCherry-tagged Ppk1. Top: Ppk1::sfGFP dendritic density is not affected by disrupting *Sec23* (these are data from the original submission). Bottom: New quantification reveals that disrupting *Sec23* does not affect the density of Ppk1 tagged with mCherry, either dual-tagged Ppk1::GFP(11)^{EC}::mCherry$^{C\text{-term}}$ or single-tagged Ppk1::mCherry. Our original quantification of the mCherry signal of the dual-tagged Ppk1::GFP(11)^{EC}::mCherry$^{C\text{-term}}$ detected a modest but significant difference from wild-type when *Sec23* was perturbed.

5) To test the effect of Rab11 on membrane localization of Ppk1, immunostaining has been carried out in Rab11 RNAi animals. It is curious that there very little staining in the cell body or axonal membrane (Fig 7A).

We thank the reviewer for pointing out our error of omitting an explanation for this immunostaining pattern. The lack of Ppk1 immunostaining on the cell bodies and axons of ddaC neurons reflects that these compartments are wrapped by glia, which exclude antibodies under non-permeabilizing conditions, such as we are using. Others have

reported a similar pattern of Ppk1 (and Ppk26) immunostaining [7,8]. We have revised the text to include this, and we apologize for our oversight in leaving out this information in the original manuscript.

New text in main text: “the anti-Ppk1 signal is absent in the proximal dendrites, cell body, and axon as these are wrapped by glia, which exclude antibodies under the non-membrane-permeabilizing conditions used to probe for membrane-localized Ppk1.”

New text in Figure 7 legend: "Under these non-permeabilizing conditions, the anti-Ppk1 antibody is unable to access the glial-ensheathed proximal dendrites, cell body, and axon."

Also, the decrease in Ppk1 levels assessed using antibody staining in Rab11 RNAi background appears to be approximately 15% (7A) whereas when assesses using Ppk1::mcherry, it seems to be much less (approximately 4%, Fig 7C). Further, the DNRab11 background, estimated decrease in Ppk1 levels based on antibody staining is approximately 40%. However, when measured using Ppk1::mcherry (7E) the decrease, though significant, is relatively modest (approximately 4% or so..). This difference is puzzling.

We thank the reviewer for these comments. Although the degree to which Ppk1 is reduced varies depending on the labeling method or the approach used to disrupt Rab11, we would like to highlight that our data consistently show that dendritic levels of Ppk1 are reduced when Rab11 function is reduced. We have taken care to probe for Ppk1 using complementary techniques (immunostaining, fluorescently tagged Ppk1) and to use different approaches to disrupt Rab11 (RNAi, dominant-negative). It is likely the degree of Ppk1 reduction depends on the method used to label Ppk1. As described in our manuscript, mCherry brightly labels Ppk1 present in internal organelles or vesicles; this differs from the membrane associated Ppk1 identified by immunostaining with anti-Ppk1 antibodies. Also, as mentioned above, we have virtually eliminated quantification of Ppk1 using mCherry-tagged Ppk1 in the revised manuscript due to the difficulty of accurately quantifying Ppk1::mCherry signal because of the bright fluorescent mCherry puncta.

It would also be interesting to know physiological effect that these changes have on the organism.

We agree that it would be interesting to know the physiological effects of manipulating Ppk1 in various ways. This is an area of active investigation but is beyond the scope of our current study.

Reviewer #3: This manuscript that is addresses the important problem of distribution of of an DEG/ENaC channnel along neuronal processes. The careful tagging of the channel with different FPs, split GFP etc are very well done. The study would have benefited from a more detailed investigation of neuronal arbor development/degeneration, transport or localization. Currently several phenotypes are briefly described.

Major:

(1) It would be nice to observe the ppk26 (& its degenerin version) localization in ppk1 mutants and vice versa, not only the phenotypic outcomes with the degenerin version of ppk26. This is particularly important as ppk1 shows epistasis only with the degenerin ppk26. Degenerin ppk26 may well act as a gain of function and localization information could potentially explain the phenotypes.

We thank the reviewer for these thoughtful suggestions. First, regarding Ppk26 localization in Ppk1 mutants (and vice versa): several independent reports have shown that Ppk1 and Ppk26 are mutually dependent on each other for their localization to the neuronal membrane [7–9]. Consistent with these reports, we also found that the membrane localization of Ppk1 depends on Ppk26 (Fig 2E).

The reviewer's suggestions led to the development of an entirely new figure that we feel enriches our story (new Fig 5). As the reviewer requested, we examined the distribution of mCherry-tagged Ppk26 in neurons under three conditions: no Ppk1, normal levels of Ppk1, or elevated levels of Ppk1. We found that the loss of Ppk1 resulted in Ppk26^{WT} and Ppk26 degenerin mutant (Ppk26^{DEG}) forming large puncta indicative of Ppk26 not reaching the neuronal membrane (new Fig 5D). This suggests that the severe dendrite arborization phenotype induced by Ppk26^{DEG} in the *Δppk1* neurons may be caused by Ppk26^{DEG} acting at an internal membrane. As described above in response to Question 2 from Reviewer 2, we found that elevating Ppk1 levels suppressed the Ppk26^{DEG}-induced dendrite arborization phenotype (the distribution of Ppk26^{DEG} in dendrites appeared similar to controls). These new data are included in the revised manuscript (new Fig 5) and below.

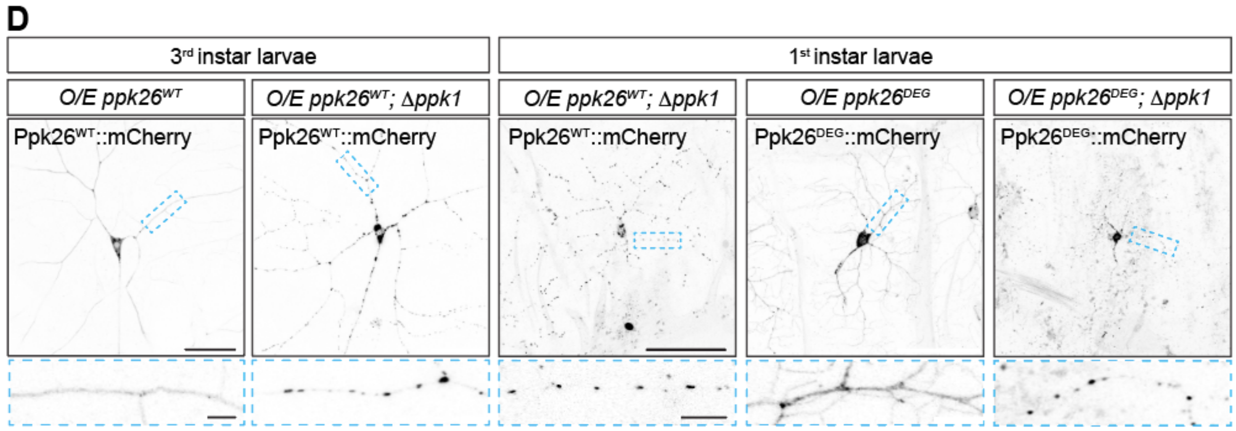

Figure 5. Effects of manipulating Ppk channel expression and activity on dendrite morphogenesis.

Text describing new results: "We attempted to suppress the dendrite arbor phenotype induced by Ppk26^{DEG} by eliminating Ppk1, which should prevent Ppk26^{DEG} from reaching the membrane. However, the loss of *ppk1* surprisingly enhanced, rather than suppressed, the Ppk26^{DEG} dendrite arborization phenotype (Fig 5C). By the 3rd larval instar stage, neurons expressing Ppk26^{DEG} and lacking Ppk1 had just cell bodies, no dendrites, and typically lacked an axon. A previous study showed that in the absence of Ppk1, Ppk26 that does not reach the membrane accumulates in large, bright puncta (Fig 5D) [9]. Similarly, we found that Ppk26^{DEG} accumulated in bright puncta in dendrites when *ppk1* was eliminated (Fig 5D; these analyses were carried out in 1st instar larvae, when the mutant neurons still occasionally had dendrites). This suggests that in the absence of Ppk1, the Ppk26^{DEG} mutant may not reach the dendritic membrane and, thus, may exert its effects on dendrite growth by acting at an internal organelle. Increasing Ppk1 levels partially suppressed the effects of Ppk26^{DEG} on dendrite growth and Ppk26^{DEG}::mCherry was normally distributed in dendrites (Fig 5E and 5F)."

With regards to the question of whether Ppk26^{DEG} acts as a gain-of-function: As mentioned in the original submission, Ppk26^{DEG} is predicted to keep the Ppk channel in an open state and thus act as a gain-of-function [7,10–12]. This gain-of-function activity likely underlies the neuronal phenotypes.

(2) Are the dendrite morphology phenotypes described due to degeneration or development? This will be useful to examine in the different genotypes that are apparently similar in their phenotypes but may actually arise from different processes being affected.

We thank the reviewer for raising this point. We revised the text and now clarify whether the dendrite growth phenotypes result from development and/or degeneration.

(3) What fraction of ppk1 overlaps with Rab11 in the cell body v/s the dendrite. Does the same hold true with Rab5. Does Rab11 localization remain changed in Rab5 RNAi?

This is an excellent suggestion. As mentioned above in response to the third question from Reviewer 1: We have attempted these experiments. However, we encountered two difficulties: first, we rarely see Ppk1::sfGFP(+) puncta. It may be that the density of Ppk1::sfGFP in transport vesicles is below our level of detection. Second, both Ppk1 and Rab11 are tagged with GFP, so we are not able to visualize their relative localization. While we have tagged Ppk1 with mCherry, we believe that the majority of the mCherry puncta that we observe reflect Ppk1 in recycling and degradative organelles (S2 Fig). Although we are not able to image Ppk1 en route to dendrites, we do see Ppk1 in the membrane of actively growing branches, throughout the branch and up to the branch tip, without discernable changes in density (Fig 3B and 3C). This is consistent with the idea that Ppk1 is integral to the membrane that drives arbor expansion.

With regards to analyzing the colocalization of Ppk1 and Rab5: again, we are prevented from examining the localization of Ppk1::sfGFP relative to Rab5 since Rab5 is tagged with YFP, which is difficult to resolve from sfGFP. We do observe colocalization between the bright Ppk1::mCherry puncta and Rab5::YFP (S2 Fig), and we found that ~half the bright Ppk1::mCherry puncta colocalize with Rab5::YFP. We also found that the number of Ppk1::mCherry puncta are significantly reduced by a dominant-negative Rab5 mutant

(S2 Fig). Combined, these data support the idea the bright mCherry puncta represent the localization of Ppk1 in a Rab5(+) organelle.

The reviewer also asks: "Does Rab11 localization remain changed in Rab5 RNAi?" We apologize, we do not quite know what the reviewer is asking with this question. We would be happy to address whether Rab11::GFP is affected by knocking-down Rab5 via RNAi, although the results of such an experiment might be difficult to interpret.

Minor comments

(1) It would be very useful to have a schematic of the neuron to decipher dendrites, axons and synapses in the images.

This is a great suggestion. We have added a schematic of a ddaC neuron that identifies the dendrites, axon, and axon terminal. We hope this schematic will help in interpreting the images (new Fig 1B).

(2) It would be useful to have membrane bound marker to conclude that ppk1 is largely on the plasma membrane in dendrites. How does one distinguish between intracellular vesicular pools from the images shown? This would benefit from some explanation.

We thank the reviewer for raising this point. Our data suggests that the split-GFP approach is an effective method for labeling membrane-associated Ppk1 (Fig 2). The distribution pattern of GFP(11)-tagged Ppk1 resembles surface-labeled Ppk1 recognized by immunostaining for Ppk1 under non-permeabilizing conditions. Also, the distribution patterns of GFP(11)- and sfGFP-tagged Ppk1 are similar. We do not know of another fluorescence-based approach that would specifically label a protein when it is membrane-bound in vivo (e.g., fluorescently tagged CD4 or CD8 also labels intracellular vesicles as do myristoylated fluorescent-protein markers).

(3) Since authors suggest that some compartments that carry ppk1 may be acidic as one reason for the difference in localization of mCherry v/s sfGFP tag. One means to test it might be to look at RNAi of a VATPase subunit which may allow one to test this hypothesis. Have the authors considered this experiment? Are the ppk1 pools intracellularly transported in an acidic environment?

This is an interesting point, and we appreciate the reviewer's thoughtful suggestions for addressing why mCherry, but not sfGFP, strongly labels vesicular and/or endosomal populations of Ppk1. We hesitate to do this experiment, however, because we do not have a good control to ensure that we would be reliably changing the pH of the vesicle or organelle in which Ppk1::mCherry fluoresces.

(4) Is the orientation of microtubules in the neurons that are used as a model established?

Previous studies (by others and our group) have revealed the orientation of microtubules in our model ddaC neurons: in axons, microtubules are uniformly oriented plus-ends-distal; in dendrites, microtubules are predominantly minus-ends-distal (this is described in the manuscript) [13,14].

Is there any contribution of microtubule polarity changes in the ppk1 localization in dynein mutants?

Disrupting dynein function does not affect dendritic microtubule polarity [15], so it is unlikely that a change in microtubule polarity affects the dendritic localization of Ppk1 when dynein function is altered. Reduced dynein function does alter axonal microtubule polarity [15], and we found that axonal Ppk1 levels are abnormally increased in the axons of *Dlic-RNAi* neurons, consistent with this previous report (S6 Fig). The change in axonal microtubule polarity likely contributes, directly or indirectly, to the change in axonal distribution of Ppk1.

Do the ectopic golgi phenotypes arise from a potential role for dynein in microtubule transport/orientation.

This is a good point, and, yes, it is likely that the mislocalization of Golgi to axons in dynein loss-of-function neurons reflects a change in microtubule-based transport [15–17]. Indeed, it is exactly because disrupting dynein affects the transport of multiple cargos and axonal microtubule polarity that we turned to the golgin Lava lamp, which connects Golgi to dynein, to selectively affect Golgi transport (S6 Fig). We found that altering Lava lamp function via a dominant-negative construct increases Ppk1 in axons similar to dynein loss-of-function, which suggests that mis-localization of Golgi alone can affect axonal density and distribution of Ppk1.

(5) When analyzing dendrite arbor phenotypes, it might be useful to divide it into primary, secondary, tertiary branches or attempt Scholl's analysis. Just measuring tips may not capture the full complexity of the dendritic arbor.

We thank the reviewer for this suggestion. We carried out Sholl analysis of the dendritic arbors of the dendrite growth mutants (see R3 Fig below). This analysis reveals that the there is a proximal shift in dendrite branches in neurons overexpressing *Rac1*. Other manipulations that affect dendrite length and branch number (*Rpl22-RNAi*, *EcR-DN*, *PI3K O/E*) do not cause a notable shift dendrite branch position. Since this analysis is consistent with previous reports and mirrors data already in the manuscript, these new data are not included in the revised manuscript.

We also carried out Sholl analysis of the arbors of *Δppk1 Δppk26* mutant neurons, and we found that there is no significant change in the distribution of branches in the neurons lacking both Ppk1 and Ppk26 (see R4 Fig below). Thus, the loss of Ppk1 and Ppk26 does not affect dendrite length, dendrite branch number, or dendrite branch patterning. Like the Sholl analysis of the dendrite growth mutants, these new data are not included in the revised manuscript (although we would be glad to add these data if the reviewer thinks they would be a meaningful addition).

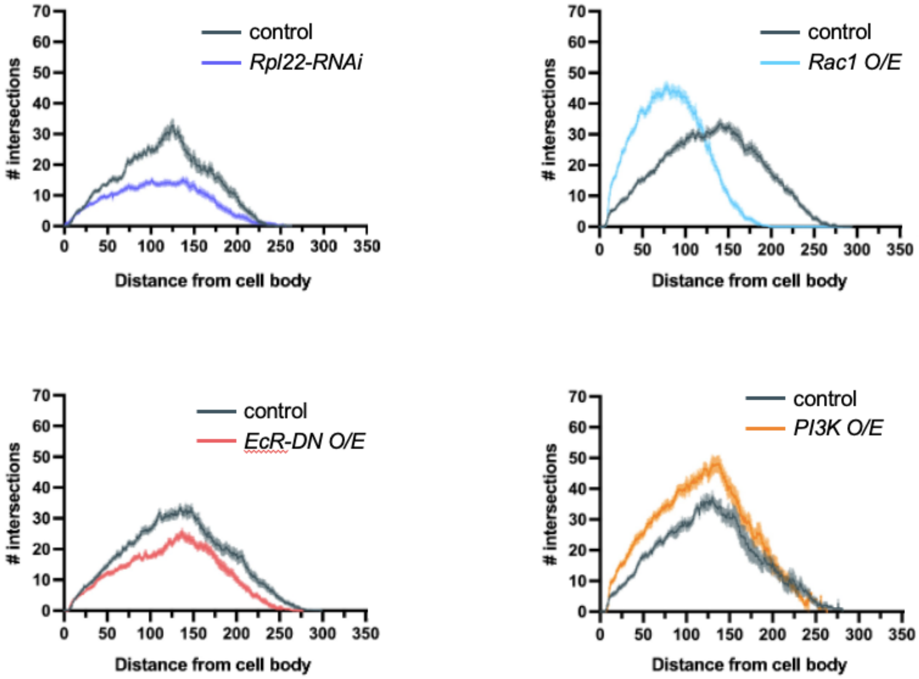

R3 Fig. Sholl analysis of dendrite branch patterning in four different conditions: knockdown of Rpl22 (*Rpl22-RNAi*) and overexpression of *Rac1*, *EcR-DN*, and *PI3K*.

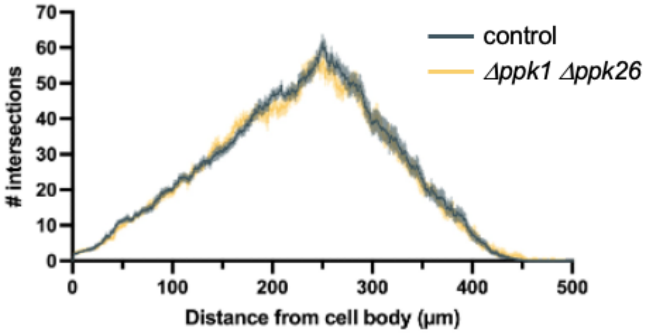

R4 Fig. Sholl analysis of dendrite branch patterning in *Δppk1 Δppk26* double-mutant neurons.

References

1. Yan C, Wang F, Peng Y, Williams CR, Jenkins B, Wildonger J, et al. Microtubule Acetylation Is Required for Mechanosensation in Drosophila. Cell Reports. 2018;25: 1051-1065.e6. doi:10.1016/j.celrep.2018.09.075

2. Tracey WD, Wilson RI, Laurent G, Benzer S. painless, a Drosophila Gene Essential for Nociception. Cell. 2003;113: 261–273. doi:10.1016/s0092-8674(03)00272-1

3. Gu P, Gong J, Shang Y, Wang F, Ruppell KT, Ma Z, et al. Polymodal Nociception in Drosophila Requires Alternative Splicing of TrpA1. Curr Biol. 2019. doi:10.1016/j.cub.2019.09.070

4. Hu C, Kanellopoulos AK, Richter M, Petersen M, Konietzny A, Tenedini F, et al. Conserved Tao kinase activity regulates dendritic arborization, cytoskeletal dynamics and sensory function in Drosophila. J Neurosci Official J Soc Neurosci. 2020; 1846–19. doi:10.1523/jneurosci.1846-19.2020

5. Krämer R, Rode S, Rumpf S. Rab11 is required for neurite pruning and developmental membrane protein degradation in Drosophila sensory neurons. Dev Biol. 2019;451: 68–78. doi:10.1016/j.ydbio.2019.03.003

6. Poe AR, Wang B, Sapar ML, Ji H, Li K, Onabajo T, et al. Robust CRISPR/Cas9-Mediated Tissue Specific Mutagenesis Reveals Gene Redundancy and Perdurance in Drosophila. Genetics. 2018;211: genetics.301736.2018. doi:10.1534/genetics.118.301736

7. Gorczyca DA, Younger S, Meltzer S, Kim SE, Cheng L, Song W, et al. Identification of Ppk26, a DEG/ENaC Channel Functioning with Ppk1 in a Mutually Dependent Manner to Guide Locomotion Behavior in Drosophila. Cell Reports. 2014;9: 1446–1458. doi:10.1016/j.celrep.2014.10.034

8. Guo Y, Wang Y, Wang Q, Wang Z. The Role of PPK26 in Drosophila Larval Mechanical Nociception. Cell Reports. 2014;9: 1183–1190. doi:10.1016/j.celrep.2014.10.020

9. Mauthner SE, Hwang RY, Lewis AH, Xiao Q, Tsubouchi A, Wang Y, et al. Balboa Binds to Pickpocket In Vivo and Is Required for Mechanical Nociception in Drosophila Larvae. Curr Biol. 2014;24: 2920–2925. doi:10.1016/j.cub.2014.10.038

10. Bianchi L. Ion Channels in Biophysics and Physiology. Adv Exp Med Biol. 2022;1349: 165–192. doi:10.1007/978-981-16-4254-8_9

11. Bianchi L, Gerstbrein B, Frøkjær-Jensen C, Royal DC, Mukherjee G, Royal MA, et al. The neurotoxic MEC-4(d) DEG/ENaC sodium channel conducts calcium: implications for necrosis initiation. Nat Neurosci. 2004;7: 1337–1344. doi:10.1038/nn1347

12. Brown AL, Fernandez-Illescas SM, Liao Z, Goodman MB. Gain-of-Function Mutations in the MEC-4 DEG/ENaC Sensory Mechanotransduction Channel Alter Gating and Drug Blockade. J Gen Physiology. 2007;129: 161–173. doi:10.1085/jgp.200609672

13. Rolls MM, Satoh D, Clyne PJ, Henner AL, Uemura T, Doe CQ. Polarity and intracellular compartmentalization of Drosophila neurons. Neural Dev. 2007;2: 7. doi:10.1186/1749-8104-2-7

14. Rolls MM. Neuronal polarity in Drosophila: Sorting out axons and dendrites. Dev Neurobiol. 2011;71: 419–429. doi:10.1002/dneu.20836

15. Zheng Y, Wildonger J, Ye B, Zhang Y, Kita A, Younger SH, et al. Dynein is required for polarized dendritic transport and uniform microtubule orientation in axons. Nat Cell Biol. 2008;10: 1172–1180. doi:10.1038/ncb1777

16. Yang SZ, Wildonger J. Golgi Outposts Locally Regulate Microtubule Orientation in Neurons but Are Not Required for the Overall Polarity of the Dendritic Cytoskeleton. Genetics. 2020;215: genetics.302979.2019. doi:10.1534/genetics.119.302979

17. Kelliher MT, Yue Y, Ng A, Kamiyama D, Huang B, Verhey KJ, et al. Autoinhibition of kinesin-1 is essential to the dendrite-specific localization of Golgi outposts. J Cell Biol. 2018;217: 2531–2547. doi:10.1083/jcb.201708096

Changes to the Original Figures

Original Fig 1 - Ppk1::mCherry data moved to new S2 Fig

Original Fig 2 - Reorganized; data on the effects of disrupting *Sec23* added (Fig 2F)

Original Fig 3 - Data on dendrite growth in the *Δppk1, Δppk26* mutant moved to new Fig 5

Original Fig 4 - Unchanged

Original Fig 5 - Revised, now new Fig 6; data on the effects of disrupting dynein activity moved to new S6 Fig.

Original Fig 6 - Figure removed and data incorporated into Fig 2

Original Fig 7 - Revised; Ppk1::mCherry data removed

Original Fig 8 - Figure removed and data incorporated into Fig 7 and new S2 Fig.

Original S1 Fig - Unchanged

Original S2 Fig - Minor reorganization, now S3 Fig.

Original S3 Fig - Revised, now S4 Fig.

Original S4 Fig - Figure removed and data incorporated into S4 Fig.

Original S5 Fig - Major revision, new data added and now new Fig 5.

Original S6 Fig - Figure removed and data incorporated into Fig 7.

Original S7 Fig - Figure and data removed.

Major additions to Figures in the revised manuscript

New Fig 5 on the effects of manipulating Ppk channel activity on dendrite growth and the localization of mutant Ppk channel.

New S5 Fig on the effects of disrupting dendrite growth on the dendritic localization of GFP::TrpA1.

New Fig 6D and 6E describe a new fly strain to express secGFP(1-10) independently of Gal4-UAS to visualize the membrane localization of Ppk1.

New Fig 7D describes the localization of Ppk1::sfGFP in *Rab11-RNAi*-expressing neurons.

New S2 Fig, panel D describes the colocalization of Ppk1::mCherry and Rab7::GFP.
