# Supplementary material for: Coordination of Pickpocket ion channel delivery and dendrite growth in Drosophila sensory neurons
Source: PLoS Genet. 2023 Nov 9;19(11):e1011025. doi: 10.1371/journal.pgen.1011025 (PMC10662761; doi:10.1371/journal.pgen.1011025)
Supplement: Attachment — Submitted filename: Mitchell et al Response Reviewers PLOS Genetics REVISED Oct.pdf [file pgen.1011025.s011.pdf]

Response to Reviewers

Reviewer #1: The authors have partially addressed my previous concerns. I still wonder whether the coordinated distribution of Ppk1 with dendritic arborization is a functional scaling or not, but I also understand the technical difficulties to answer my previous comments. Thus, I have no further comments to the submitted manuscript.

We thank the reviewer for their constructive comments during the review process, which have significantly improved our manuscript.

Reviewer #2: The authors have generated tools to study the mechanisms regulating the trafficking of Ppk1 into dendrites of DDa neurons by generating different tagged versions of the protein. They have tested these tools rigorously to demonstrate that they can be used reliably to monitor localization and trafficking of Ppk1. Using these reagents the authors show that Ppk1 channel are present from the very early stages of dendtrite formation. The levels of these channels (normalized to membrane area) is relatively constant and does not change in response to altered dendrite morphology; these channels do not diffuse rapidly in the membrane. Trafficking to the dendrites is via golgi and golgi outposts and is dependent on Rab11. Loss of Rab11 leads to decrease in channel levels at the membrane. Ppk1 is required for membrane localization of Ppk26DEG: loss of Ppk1 enhances the arborization phenotype of Ppk26DEG. This is an improved version of the manuscript. The concerns raised earlier have been addressed. The authors have also developed and used an improved version of the split GFP system in their study.

We thank the reviewer for kindly appreciating the improvements to the revised manuscript and thank them for their earlier suggestions, which led to this improvement. We are glad that the previous concerns have been addressed.

(i) One major concern here is the opposing results seen upon perturbing dynein function. The authors show that disrupting dynein activity does not have an immediate effect on Ppk levels in the membrane. But, over time (in a 3rd instar larva) there is a decrease in channel levels measured using Ppk1-sfGFP levels.
However, they see the opposite with the split GFP system ie. there is an accumulation of Ppk1 in the membrane. Interestingly, immunostaining using non-detergent conditions shows no change. The authors suggest that the increase in intensity observed with the split GFP system compared with immunostaining is likely due to increased sensitivity. However, they do not explain why the result is opposite to what is seen with Ppk1-sfGFP. In the discussion they state that the increase in intensity seen with the split-GFP system is likely due to poor turnover of the Ppk1 protein and they use the slow diffusion rates seen in FRAP to support this conclusion. However, this argument is rather weak. Not sure if FRAP can be used to comment on protein stability at the membrane.

We very much appreciate the reviewer's comments about reconciling differences in dendritic Ppk1 density when using different approaches to visualize Ppk1 in neurons with reduced dynein function. We propose the following model as a potential explanation of these results, and we welcome any additional feedback the reviewer may have to explain these discrepancies. We reason that differences between Ppk1::sfGFP and Ppk1::GFP(11)^{EC} likely reflect that Ppk1::sfGFP reports Ppk1 that is present in both the outer cell membrane and internal membranes, whereas Ppk1::GFP(11)^{EC} reports just Ppk1 localization on the cell membrane. We interpret these results to suggest that the loss of dynein activity over time may reduce Ppk1 that is present on internal membranes, such as transport vesicles that supply Ppk1, but may not affect Ppk1 that has already been delivered to the dendritic membrane (see also new S8 Fig, generated in response to a comment by Reviewer 3). Thus, the decrease in dendritic Ppk1::sfGFP density over time

likely reflects a decrease in Ppk1 delivery. In contrast, dynein does not affect the density of Ppk1 that has already been delivered and inserted into the cell membrane. We have attempted to clarify this model in a revised summary of these findings in the text, lines 377-379 (see also Minor concern ii below.) We are not sure what an *increase* in Ppk1::GFP(11)^{EC} fluorescent signal may reflect –it may be a secondary consequence of the unknown mechanism by which the dendritic arbor degenerates as dynein function decreases over time.

We also thank the reviewer for mentioning that FRAP is not typically used to assess the stability of proteins inserted into the cell membrane, and we have removed reference to the FRAP data from this part of the Discussion (line 476).

Minor concern
(ii) Lines 372-373: 'Our results also suggest that while overall levels of Ppk1 decrease when 373 dynein function is perturbed over time (e.g., Ppk1::GFP; Fig 6A), Ppk1 that does make it into 374 dendrites and the dendritic membrane is not diminished by reduced dynein activity.' Not sure what is meant here.

We have revised this sentence in an attempt to clarify our meaning (lines 377-379 in the revised manuscript): "Our results also suggest that while the overall dendritic levels of Ppk1 decrease when dynein function is perturbed over time (e.g., Ppk1::GFP; Fig 6A), Ppk1 that does make it into the dendritic membrane is not reduced by diminished dynein activity."

Reviewer #3: Authors have significantly improved the manuscript.

The study attempts to understand the trafficking of the sensory neuron ion channel in one class of Drosophila sensory neurons. The strength is the manuscript is the nice split-GFP system. The weakness is the lack of any real mechanistic insight. However, the study is well worth publishing, is well done and significantly improved since the earlier submission. Several things of note: very few systematic studies that address trafficking of an endogenous channel in vivo which this study does, the assessment of the role of ppk26 (old problem how do multiple proteins of the same complex help in targeting), phenotypic roles in development of the dendritic arbor.

We thank the reviewer for highlighting strengths of our study and supporting its publication.

I have a few minor suggestions which I hope will improve the manuscript.

It would be good if authors mention & discuss the possibility that "mis-localization of Golgi alone can affect axonal density and distribution of Ppk1" that their data suggest and they themselves say in the reviewer response document.

We thank the reviewer for this suggestion, and we have incorporated the following text in the revised manuscript (lines 345-347):

"[…] disrupting the Golgi-dynein adaptor Lava lamp also resulted in ectopic Golgi outposts in axons and increased axonal Ppk1::sfGFP, which suggests that mis-localization of Golgi alone can affect the axonal density and distribution of Ppk1."

I also think it would be useful to add the Sholl analysis in the supplement given this analysis method is widely used.

We are happy to include the Sholl analysis: see new panel in Fig 5A and new figure S5 Fig.

Finally, a diagram of how authors envisage this trafficking works would not be amiss.

This is a nice suggestion. See new figure S8 Fig.

Have all data underlying the figures and results presented in the manuscript been provided? Large-scale datasets should be made available via a public repository as described in the *PLOS Genetics*data availability policy, and numerical data that underlies graphs or summary statistics should be provided in spreadsheet form as supporting information.

Reviewer #1: Yes

Reviewer #2: Yes

Reviewer #3: **No:** I think excel sheets of some of the data should ideally be provided. I was not able to find it.

Virtually all the graphs that report our data include individual dots that indicate the numerical data used for quantification and statistical analysis. There is one graph, the representation of the FRAP experiment results (Fig 3D), that does include individual data points, and we have added a spreadsheet with these data (new S1 Table).

PLOS authors have the option to publish the peer review history of their article (what does this mean?). If published, this will include your full peer review and any attached files.

If you choose "no", your identity will remain anonymous but your review may still be made public.

Do you want your identity to be public for this peer review? For information about this choice, including consent withdrawal, please see our Privacy Policy.

Reviewer #1: No

Reviewer #2: No

Reviewer #3: No
